# Supplementary material for: The smfBox is an open-source platform for single-molecule FRET
Source: Nat Commun. 2020 Nov 6;11:5641. doi: 10.1038/s41467-020-19468-4 (PMC7648814; doi:10.1038/s41467-020-19468-4)
Supplement: Supplementary file 1 — Supplementary Information [file 41467_2020_19468_MOESM1_ESM.pdf]

Supplementary Information: The smfBox is an open-source platform for single-molecule FRET

*Benjamin Ambrose[1†], James M. Baxter[1†], John Cully[1†], Matthew Willmott[1], Elliot M. Steele[2], Benji C. Bateman[3], Marisa L. Martin-Fernandez[3], Ashley Cadby[2], Jonathan Shewring[1], Marleen Aaldering[1], Timothy D. Craggs[1]**

[1] Sheffield Institute for Nucleic Acids, Department of Chemistry, University of Sheffield, Sheffield, UK
[2] Department of Physics, University of Sheffield, Sheffield, UK
[3] Central Laser Facility, Research Complex at Harwell, Rutherford Appleton Laboratory, Oxford, UK
[†] *Equal contribution.* **Corresponding author: t.craggs@sheffield.ac.uk*

Supplementary Material	Location
**Supplementary Note 1**<br>Hardware construction and alignment	pg 2-8
**Supplementary Note 2**<br>C++ Software - smOTTER	pg 9-14
**Supplementary Note 3**<br>LABVIEW Software	pg 15-16
**Supplementary Note 4**<br>Data format	pg 16
**Supplementary Note 5**<br>DNA sequences	pg 17
**Supplementary Note 6**<br>Accurate FRET validation	pg 18
**Supplementary Note 7**<br>Asymmetric ALEX	pg 19-23
**Supplementary Note 8**<br>Verification of Dynamics	pg 24
**Supplementary Note 9**<br>Possible Expansions	pg 25
**Supplementary Note 10**<br>FCS, FCCS	pg 26
**Supplementary Figures**	pg 27-38
**Supplementary Equations**	pg 39
**Supplementary References**	pg 40
**Supplementary Data 1**	AutoCAD_Assembled_smfBox.dwg
**Supplementary Data 2**	Technical_Drawings_smfBox.dwg
**Supplementary Movie 1**	smfBox.mp4

GitHub: https://craggslab.github.io/smfBox/

Latest acquisition software: smOTTER and LABVIEW

Technical drawings: aluminium parts and 3D AutoCAD Model

Raw data and analysis including Jupyter Notebooks, HDF5 Files, and PAM Files, (also available at: https://zenodo.org/record/3625603#.XxmB3ChKhm9

Supplementary Note 1: *Hardware construction and alignment*

Here we provide detailed schematics and instructions for the assembly of the smfBox (single-molecule FRET box – pronounced Smurf Box). The smfBox can be built in three parts, (1) the excitation pathway, (2) the aluminium box and its contents, and (3) the emission pathway (Fig N1.1). Here we provide an overview of the components for each part, building and alignment instructions, and AutoCAD files of the final assembled microscope (Supplementary Data 1: AutoCAD_Assembled_smfBox.dwg) and technical drawings (Supplementary Figures 1-14 and Supplementary Data 2: Technical_Drawings_smfBox.dwg). The most recent build instructions are available at our GitHub, https://craggslab.github.io/smfBox/.

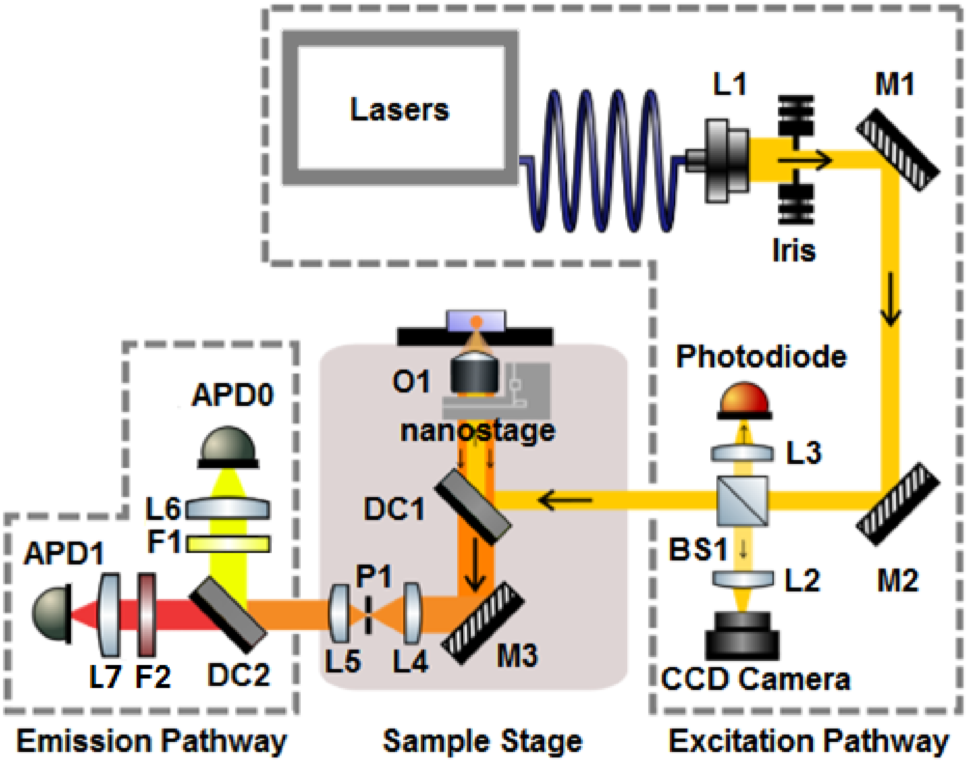

Supplementary Fig. N1.1: 2D Schematic of the smfBox showing the Excitation pathway, box, and emission pathway.

1.1 Excitation Pathway

The excitation pathway is built according to Supplementary Fig. N1.2, from a precision iris, two kinematically mounted mirrors, a beam splitter, a photodetector, and a CCD camera. Optomechanical components are mounted on non-height adjustable posts with cage-rods between them to ensure robustness of alignment. After being cropped by the iris, the mirrors M1 and M2 give complete axial stabilisation of the beam before entering the box. ND filters can be inserted between mirrors M1 and M2 to attenuate the laser power if required. The beam splitter BS1 on the first pass reflects 10% of light through a lens (L3) onto a nanosecond rise-time photodetector for calibration of laser alternation and power, whilst permitting the other 90% through to the cube. Backscattered light from the sample is reflected off the back of the beam splitter, through a lens (L2) onto a CCD camera which is used for focusing the objective. One potential problem can arise from reflections off the photodetector passing through the beam splitter onto the CCD (dashed red line), this is averted by mounting the photodetector at a slight angle.

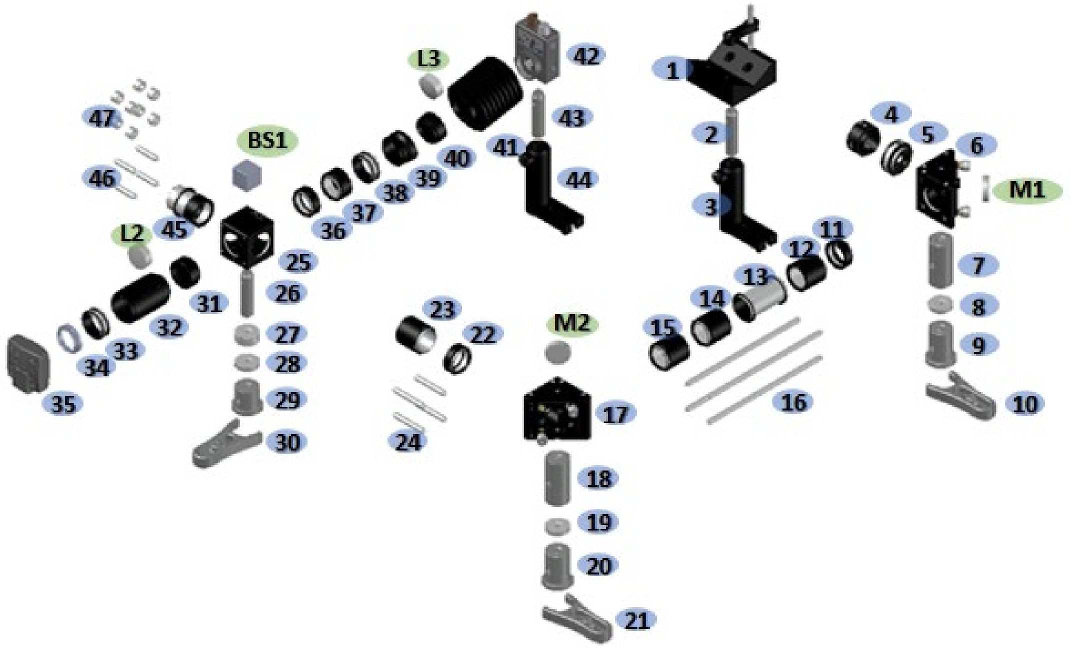

Supplementary Fig. N1.2: Exploded view of the excitation path. Parts are labelled according to Supplementary Table N1.1.

Supplementary Table N1.1: Excitation path components

Part Nr	Product Code	Use	Part Nr	Product Code	Use
1	VC3C/M	V-shaped collimating lens holder	27	RS10/M	Post spacer BS1 (L=10mm)
2	TR50/M	Collimating lens post	28	RS5/M	Post spacer (L=5mm)
3	UPH50/M	Post holder	29	RS1P8E	Pedestal Pillar Post BS1 (L=1'')
4	SM1P1	Attachment lens to iris	30	CF125C/M	Clamping fork for BS1 post
5	SM1D12C	Iris	31	SM1L03	Tube 1 BS1 to DCC
6	KCB1C/M	Kinematic Mirror Mount for M1	32	SM1L20C	Slotted lens tube for L2
7	RS50/M	Pillar post (L=50 mm)	33	SM1T2	Tube coupler BS1 to DCC
8	RS5/M	Post spacer (L=5mm)	34	SM1A9	Adapter SM1/C-mount BS1 to DCC
9	RS1.5P/M	Pedestal pillar post (L=38mm)	35	DCC1545M	CCD Camera
10	CF125C/M	Clamping fork for M1 post	36	SM1L03	Tube 1 BS1 to photodetector
11	SM1L03	Tube 1 M1 to M2	37	SM1L05	Tube 2 BS1 to photodetector
12	SM1L10	Tube 2 M1 to M2	38	SM1T2	Tube coupler BS1 to photodetector
13	SM1T20	Tube coupler M1 to M2	39	SM1L03T	Angled tube BS1 to photodetector
14	SM1L10	Tube 3 M1 to M2	40	SM1L03	Tube with L3
15	SM1L10	Tube 4 M1 to M2	41	SM1B3	Tube Bellow BS1 to photodetector
16	ER6-P4	4x rods M1 to M2 (6'')	42	DET10A/M	Photodetector
17	KCB1C/M	Kinematic Mirror Mount for M2	43	UPH50/M	Post holder
18	RS50/M	Pillar post (L=50 mm)	44	TR50/M	Post for photodetector
19	RS5/M	Post spacer (L=5mm)	45	SM1V10	Tube BS1 to box
20	RS1.5P/M	Pedestal pillar post (L=38mm)	46	ER1-P4	4x Cage Rods BS1 to box
21	CF125C/M	Clamping fork for M1 post	47	ERSCA-P4	2x4 Rod adapter BS1 to box
22	SM1L03	Tube 1 M2 to BS1	M1	BB1-E02	First Mirror
23	SM1S10	Tube 2 M2 to BS1	M2	BB1-E02	Second Mirror
24	ER1.5-P4	1x4 rods M2 to BS1 (1.5'')	BS1	21012	10/90 Beam splitter
25	CM1-DCH/M	Cage Cube for BS1	L2	AC254-030-AML	Lens for Camera
26	TR50/M	Post for BS1(L=50mm)	L3	49793	Lens for Photodetector

1.2 The Box

The smfbox does not use a commercial microscope body. Instead, the excitation dichroic, objective, and sample stage are housed in an anodised-aluminium box (Supplementary Fig. N1.3). After machining, anodising, and tapping, the box parts are assembled: Initially, part A is screwed to the optical table, followed by G, E and F. Then the optical components are assembled (Supplementary Fig N1.4), and attached to F. Then the box can be completed, with the attachment of parts C, H and I. The final step involves construction and attachment of the lid (J) and shutter mechanism (D).

Note that the smfBox is originally designed for a metric optical breadboard (25 mm spacings). For imperial or any other spacing, the holes in the base plate (A) for attachment to the optical table will have to be machined differently (Fig. S4).

Supplementary Fig. N1.3: An exploded view of the aluminium box. Parts are labelled according to Supplementary Table N1.2.

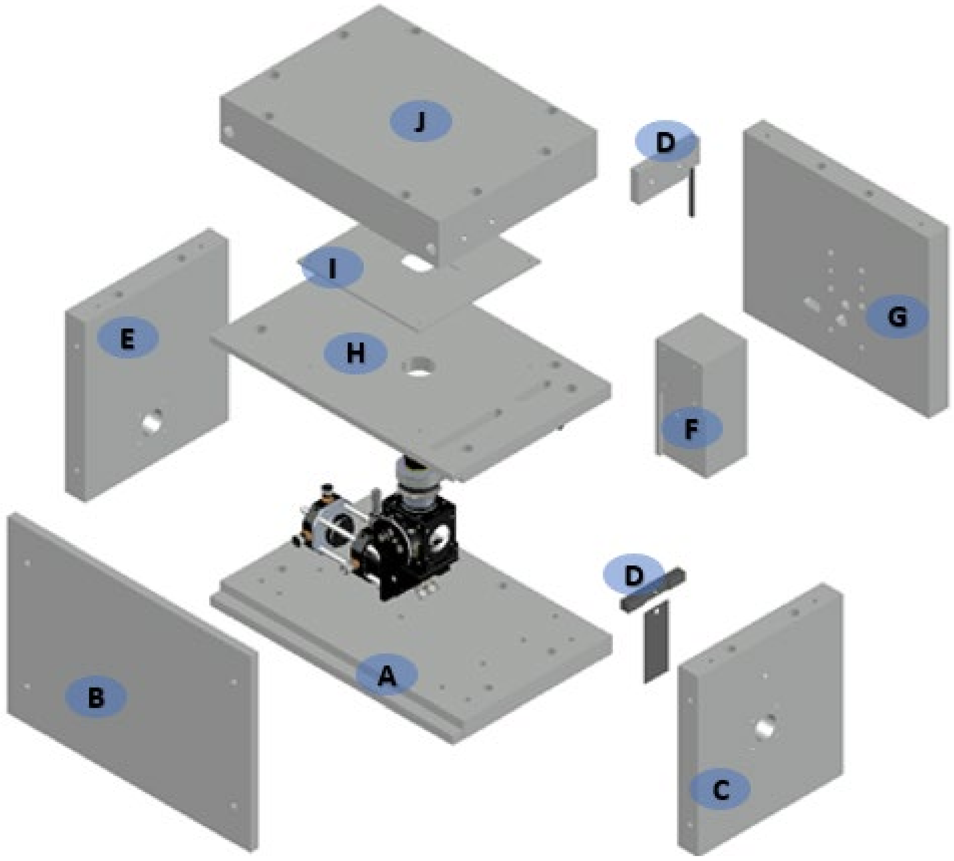

Supplementary Table N1.2: Aluminium Box components. See technical drawing in supplementary figures for engineering diagrams (or files online at our GitHub).

Part Nr	Use	Figure	Part Nr	Use	Figure
A	Base Plate	S4	F	Block	S5
B	Front Plate	S6	G	Back Plate	S3
C	Laser-In Plate	S7	H	Top Plate	S12
D	Shutter	S10	I	Stage	S14
E	Laser-Out Plate	S8	J	Lid	S9

Upon entering the box, lasers are reflected by the excitation dichroic DM1 into the objective and sample. The objective can be moved through z by a nanostage. The sample stage is static, but the design is compatible with an xy nano-stage if required (allowing for potential applications in confocal scanning techniques). Emitted light from the sample then passes back through the objective and is permitted through DM1, and onto a broadband mirror M3. Lens L4 then focuses emitted light through a pinhole P1 to remove out of focus light before reaching a second lens L5 (shown in emission pathway). L4 can be adjusted in xy, P1 has full adjustment in xyz and L5 is static.

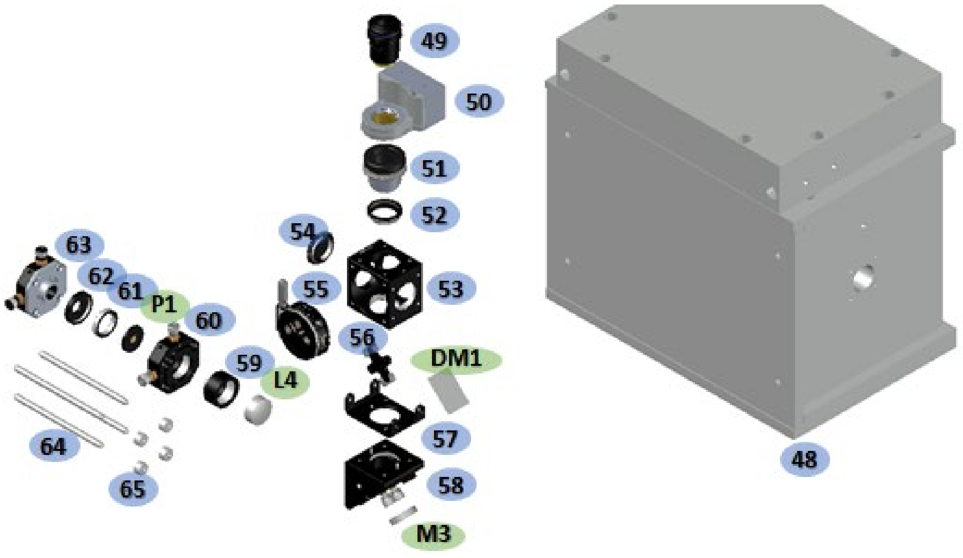

Supplementary Fig. N1.4: An exploded view of the optical components inside the microscope body, with parts labelled according to Supplementary Table N1.3.

Supplementary Table N1.3: Part numbered microscope body components.

Part Nr	Product Code	Use	Part Nr	Product Code	Use
48	N/A	Machined Aluminium Box	59	SM1L05	Lens Tube L4 (0.5'')
49	N1480700	Objective	60	CXY1	Translating Lens Mount for L4
50	FOC.300	Single axis nanostage (Piezoconcept)	61	SM1A3	Adapter (Ext. SM1 Int. RMS) for P1 to Mount
51	N/A	Piezoconcept bushing (with nanostage)	62	SM1A1	Adapter (Ext. SM05 Int. SM1) for P1 to Mount
52	SM1A12	FOC bushing adapter (Ext. SM1 Int. M25)	63	CXYZ05/M	Translating Mount for P1
53	C4W	Cage Cube DM1	64	ER4-P4	1x4 4'' Rods
54	SM1CP2	Cap for spare DM1 cage cube ports	65	ERSCA-P4	1x4 Rod Adapter
55	B4CRP/M	Precision mount for DM1	DM1	ZT532/640rpc	Excitation Dichroic
56	FFM1	Clamp for DM1	M3	MM3-311-t6-1 25.4	Hard Mirror between DM1 and L4
57	C4W-CC	Cage Cube Connector M3 to DM1	L4	Edmund - 49792	Lens before pinhole (50 mm FL)
58	KCB1/M	Right angle KM mount for M3	P1	PNH-20	Pinhole (20 µm)

1.3 The Emission Pathway

The emission pathway is constructed as in Supplementary Fig. N1.5. The first dichroic mirror in the emission pathway, DM2, reflects light below 640 nm, sending donor emission into the path to APD0 whilst permitting acceptor photons through to APD1. In both cases a band-pass filter (F1, F2) is used to clean up laser bleed-through and Raman scatter from the emission, and a lens (L6, L7) is used to focus the beam onto the APD. The optical properties of the smfBox are summarised in Supplementary Fig. N.6.

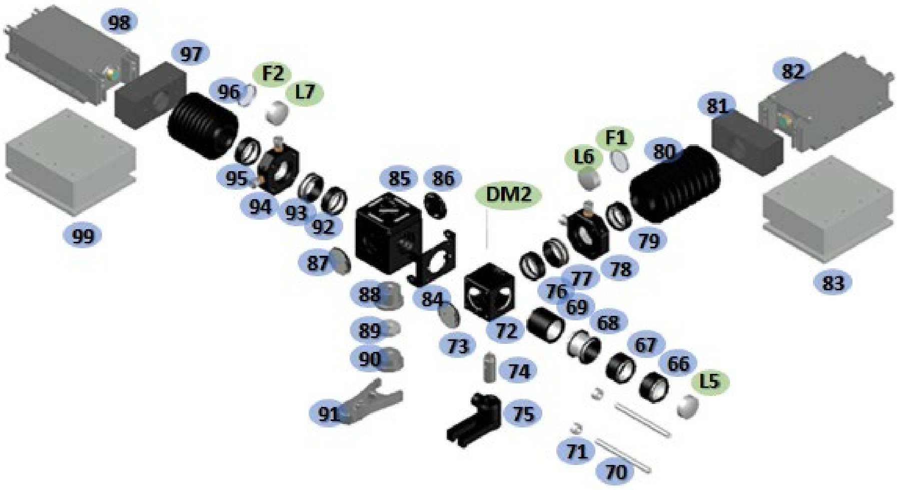

Supplementary Fig. N1.5: An exploded view of the Emission pathway with components labelled according to Supplementary Table N1.4.

Supplementary Table N1.4: Emission path components. Technical drawings for the APD cover and mount are provided in Supplementary Figures (shown in brackets in the table) and on our GitHub.

Part Nr	Product Code	Use	Part Nr	Product Code	Use
66	SM1L05	Tube 1 box to DM2 with L5	86	SM1CP2	Caps for spare ports of filter cubes
67	SM1L05	Tube 2 box to DM2	87	SM1CP2	
68	SM1T10	Tube coupler box to DM2	88	RS075P/M	Pedestal pillar post (L=19mm)
69	SM1L10	Tube 3 box to DM2	89	RS5/M	Spacer (L=5mm)
70	ER3	2x Rods box to DM2 3''	90	RS05P	Pedestal pillar post (L=0.5'')
71	ERSCA	2x Rod adapters box to DM2	91	CF125C/M	Clamping fork filter cube
72	CM1-DCH/M	Cage cube for DM2	92	SM1L03	Tube 1 DM2 to APD0
73	SM1CP2	Cap for spare port of DM2 cage cube	93	SM1T2	Tube adapter DC2 to APD1
74	TR30/M	Post DM2 (L=30 mm)	94	CXY1	Translating lens mount
75	UPH30/M	Post Holder DM2 (L=30mm)	95	SM1L03	Tube for F2 DC2 to APD1
76	SM1L03	Tube 1 DM2 to APD0	96	SM1B3	Lens tube bellow DC1 to APD1
77	SM1T2	Tube adapter DM2 to APD0	97	APD Cover **(S1)**	Connector tube to APD1
78	CXY1	Translating lens mount	98	SPCM-AWRH-14	APD1 (Excelitas)
79	SM1L03	Tube for F1 DM2 to APD0	99	APD Mount **(S2)**	Post APD1
80	SM1B3	Lens tube bellow DM1 to APD0	L5	Edmund - 49793	Lens after Pinhole (63.5 mm FL)
81	APD Cover **(S1)**	Connector tube to APD0	L7	AC254-75-B-ML	APD1 Lens
82	SPCM-AWRH-14	APD0 (Excelitas)	L6	AC254-100-A-ML	APD0 Lens
83	APD Mount **(S2)**	Post APD0	F2	FF01-679/41-25	Acceptor clean-up filter
84	CM1-CC	connector DM2 to filter cube	F1	FF01-571/72-25	Donor clean-up filter
85	DFM1/M	Fluorescence filter cube	DM2	NC395323-T640lpxr	Emission Dichroic

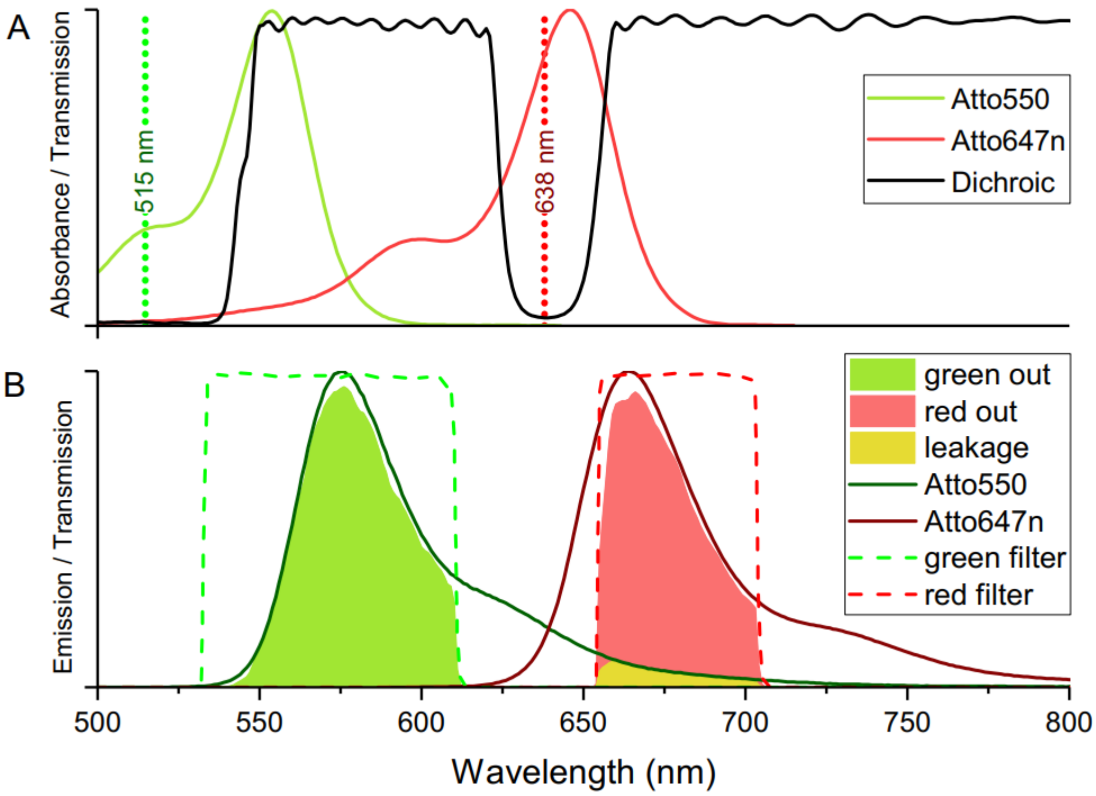

Supplementary Fig. N1.6: Spectra of optical components in the smfBox. **a:** The excitation dichroic in black with laser lines and typical dye absorption spectra in green and red. **b:** The two emission channels shown in dashed lines, with typical emission spectra. Block colours show the spectra of emission which ultimately reaches the detectors. An FPbase profile is available at https://www.fpbase.org/microscope/Aa5VpxP9ff3nmsdjnYBRUb/

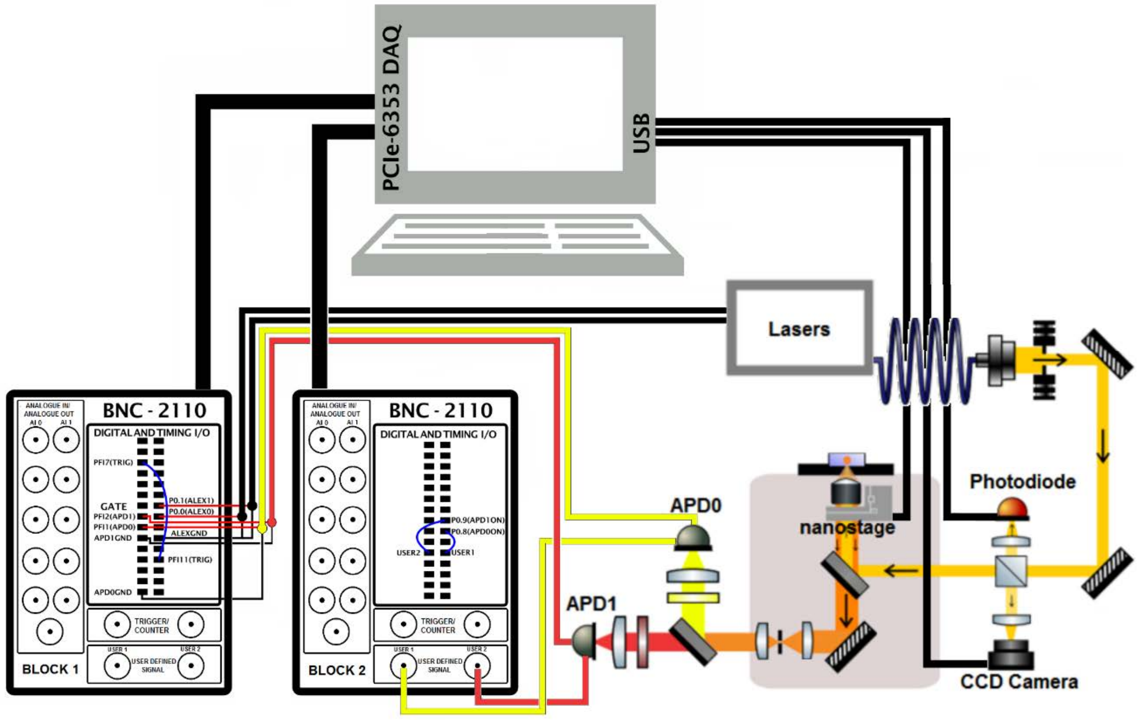

Supplementary Fig. N1.7: Wiring diagram of electronics in the smfBox. Lasers are controlled, and APD's are gated and monitored via BNC adaptors connected to the NI-DAQ board in the PC. The CCD camera and nanostage for Z focussing, as well as the photodiode for laser power monitoring are connected directly via USB.

1.4 Alignment

Once the main cube body is attached the optical table the scope can be built and aligned (~ 2 days work).

1. Once the main excitation pathway: M1, M2 and BS1 have been mounted they should be connected with 3 of the 4 cage rods without the lens tubes attached. The initial alignment can be done by aiming the beam to the centre of the DM1 mount port, using a target on one of the dichroic cube's mount caps. A handy trick to give fine alignment is to rotate DM1 to be 180° to the incoming beam and place a mirror directly behind it. This mirror will reflect the bleed-through of the dichroic, creating a cavity. If the beam is flat and correctly aligned coming into the dichroic mount then the reflection of the beam should match the incoming one.

2. Insert the fourth rod and the lens tubes and repeat the above steps to give fine alignment.

3. The microscope body is then aligned without insertion of the objective, nanostage or confocal system (L4, L5 and P1): DM1 should then be aligned at 45° to reflect the beam perpendicular to the plane of the optical table (aiming to be centred on the objective's aperture when inserted). To check this alignment long rods can be extended from the DM1 mount with the top lid of the microscope body removed. Using two frosted glass apertures or collapsible irises, the alignment can be tuned as the beam should stay in the centre of both apertures, monitored with a power meter. Adjustment of the pitch of DM1 should correct for any deviations. Alternatively, mirrors M1 and M2 can be used to obtain a purely vertical, and centred excitation beam, again monitored by a power meter.

4. Alignment of the confocal system should then be done by inserting a mirror in the position of O1. With the laser power increased the bleed-through should be visible though the dichroic. By using long rods and collapsible irises attached to the beam-out port of the microscope body M3 can be aligned to be flat and centred.

5. The confocal lenses, L4 and L5 can then be positioned by checking the beam is collimated at a long distance. This can be done by measuring the beam waist with a camera or through visual inspection and adjusting the position of the lenses relative to each other.

6. The pinhole can then be inserted and aligned using a power meter to track the maximum transmission.

7. The objective lens can then be inserted, and the top plate of the box attached. Using a microscope slide with a drop of water the coarse focusing of the objective lens can be found using the operational software (see below).

8. Using an appropriate dye (eg. Cy3B) the pinhole alignment can then be optimised further using a power meter.

9. If the dye concentration is high enough the excitation pathway can then be coarsely aligned by-eye without the bellows attached. For fine alignment, the dye concentration or laser power can be reduced. The translation stages on the APD lenses (L6 and L7) can then be used with the APD alignment software to get exact alignment.

10. The bellows should then be attached, and the system is ready for testing.

Supplementary Note 2: *Operational Software* – smOTTER

We provide operational software in the form of both a standalone C++ implementation called smOTTER, and a set of LABVIEW VI's which can be more easily customised.

2.1 Installation

Pre-compiled executables are available from our github or the supplementary materials of this paper. To install, simply download the zip, extract it to a location of your choosing and run the smfBoxAcquisition.exe.

The executables require the following to be installed to operate correctly:

- A copy of NI-DAQmx
- A 64-bit copy of the ThorCam software

2.2 Compilation from Source

A copy of Visual Studio 2017 or later and an installation of Qt 5.12 is required to compile from source. The code additionally depends on the NIDAQ C SDK which comes with an installation of DAQmx, the ThorCam C SDK which comes with an installation of the ThorCam software and a copy of the HDF5 C++ library. The HDF5 library can be compiled from source (Version 1.10.4 was used to develop the software) or a compatible compiled version can be downloaded from our GitHub.

Once all the dependencies have been installed, download the source code. The project can now be opened in Qt Creator (installed by default with Qt). Alternatively, the project can be opened in Visual Studio by installing the Qt plugin and selecting import .pro file project from the Qt VS dropdown. The code can then be compiled by selecting the build (or build and run) button in your chosen IDE. The executable should appear in %SRC_DIR%/build/Debug or %SRC_DIR%/build/Release depending on your compilation settings

2.3 User Interface

The typical procedure for taking data with smOTTER is as follows; immersion oil and a coverslip is placed on the objective of the smfBox, a sample (10-100 µl) is applied and the lid is closed. The "z-focus" tab is used to position the z-stage such that the confocal volume is in the sample (see Supplementary Figure N2.6 for detail). Note that on slower computers the live trace may be slowed with the camera on, so the camera is stopped before acquisition. Then in the "live trace" tab, pressing the "live" button in the bottom left will begin displaying live data without saving. This is used to determine if the sample is at an appropriate concentration (1~ burst per second). If the sample is to be changed before acquisition, "stop" is pressed before opening the lid of the smfBox. When ready to acquire and save data, the experiment length and save frequency is entered, along with all save settings. On pressing "start", smOTTER will begin to save acquired data.

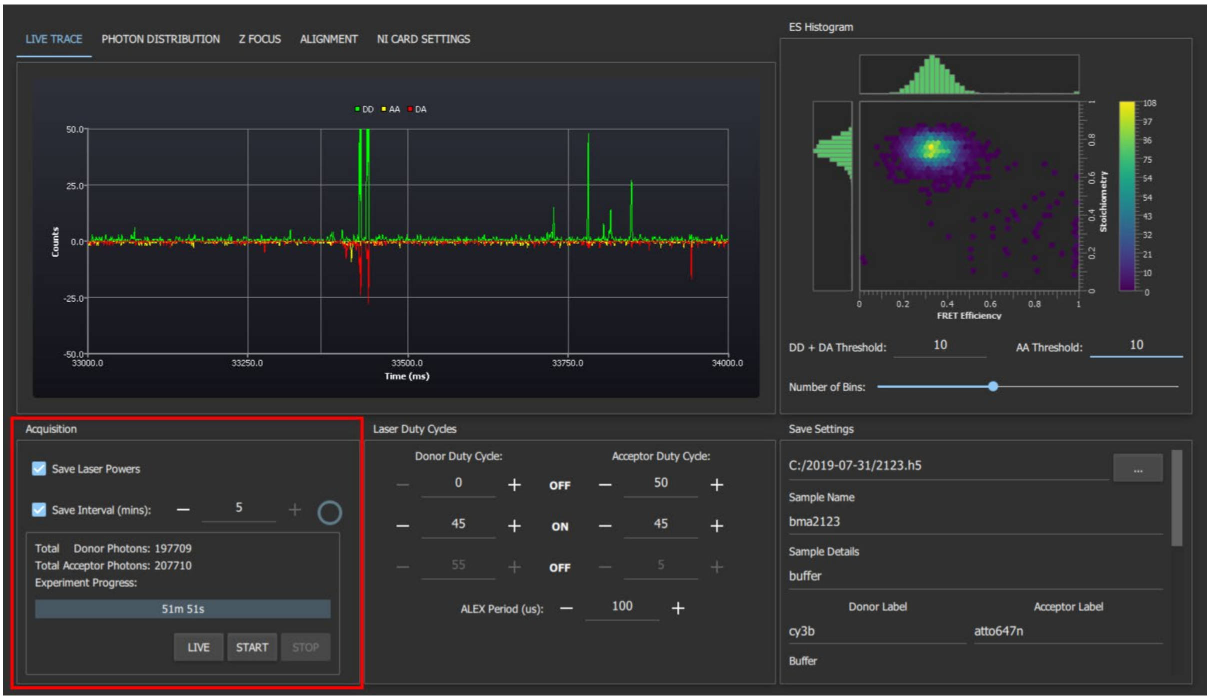

Supplementary Fig. N2.1: The acquisition settings section contains settings for and status of the current experiment. The experiment length setting controls the time the experiment should run for. The save laser powers setting can be used to perform real time recording of the laser powers as reported by the system's laser power photo-detector. The save interval checkbox toggles automatic saving data to disk during acquisition. The save interval can also be set to the desired value using the field next to the checkbox. During acquisition the circle to the right of the save interval settings indicates the time until the next save to disk. Underneath the acquisition settings is a box containing the total number of donor and acceptor photons collected, the experiment progress indicator, and the acquisition control buttons. The start and stop buttons begin and abort an acquisition respectively. The live button starts an acquisition but does not save anything to disk. This is useful for checking that everything is set up correctly before starting the acquisition.

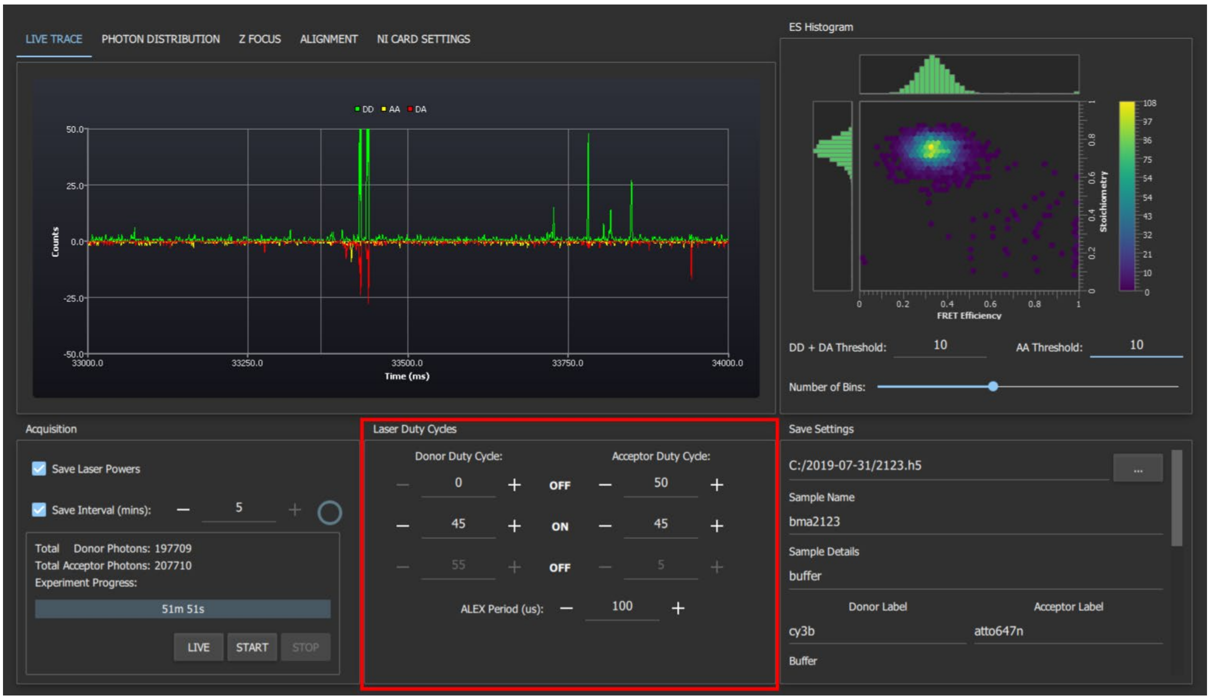

Supplementary Fig. N2.2: The laser duty cycles section allows configuration of the laser alternation periods. The first "off" row sets the time from the beginning of an ALEX period that the corresponding laser remains off for. The "on" row sets the time that the lasers remain on for. The final "off" row is the time remaining at the ALEX period that the lasers will be off for. All timings are in percentages of the ALEX period. A graphical representation of the laser duty cycles is available in the "photon distribution tab". The final setting in this section is the ALEX period in us. This is usually set to 100 us as this allows for exact synchronisation between all the functions of the NIDAQ board.

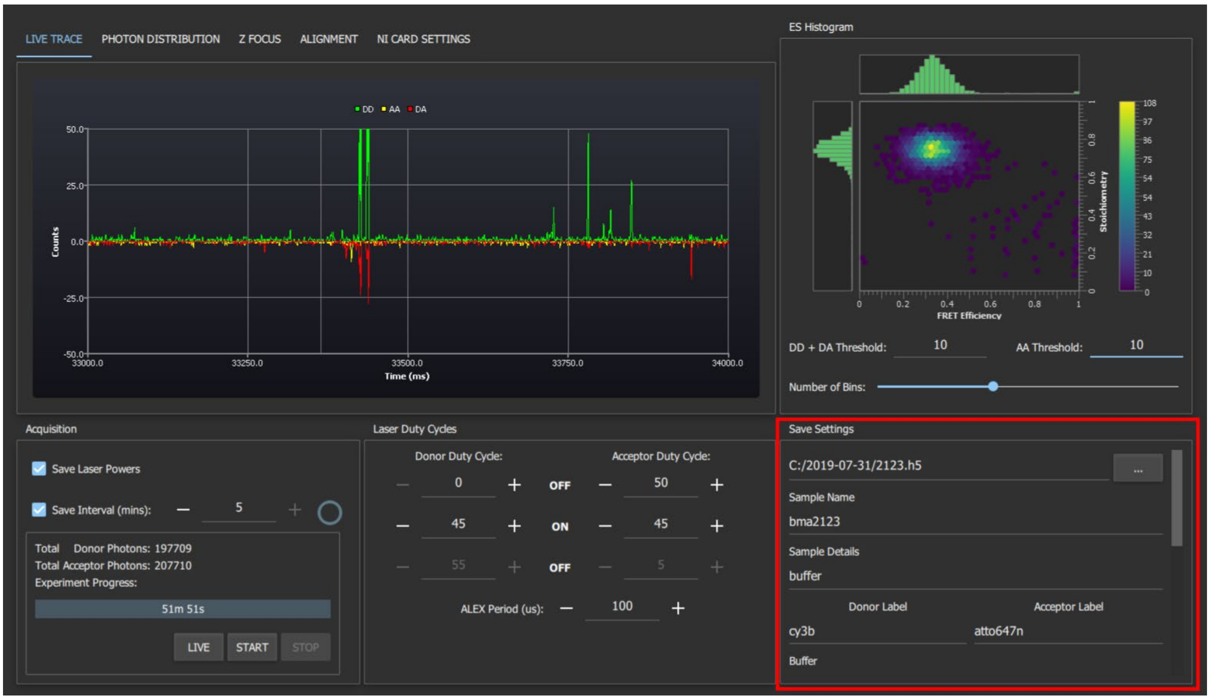

Supplementary Fig. N2.3: The save settings box contains inputs for all the metadata required by PhotonHDF5. Settings that are unlikely to change between experiments, e.g. laser wavelengths, are remembered between restarts of the program. The program will ask you before overwriting any previously saved data.

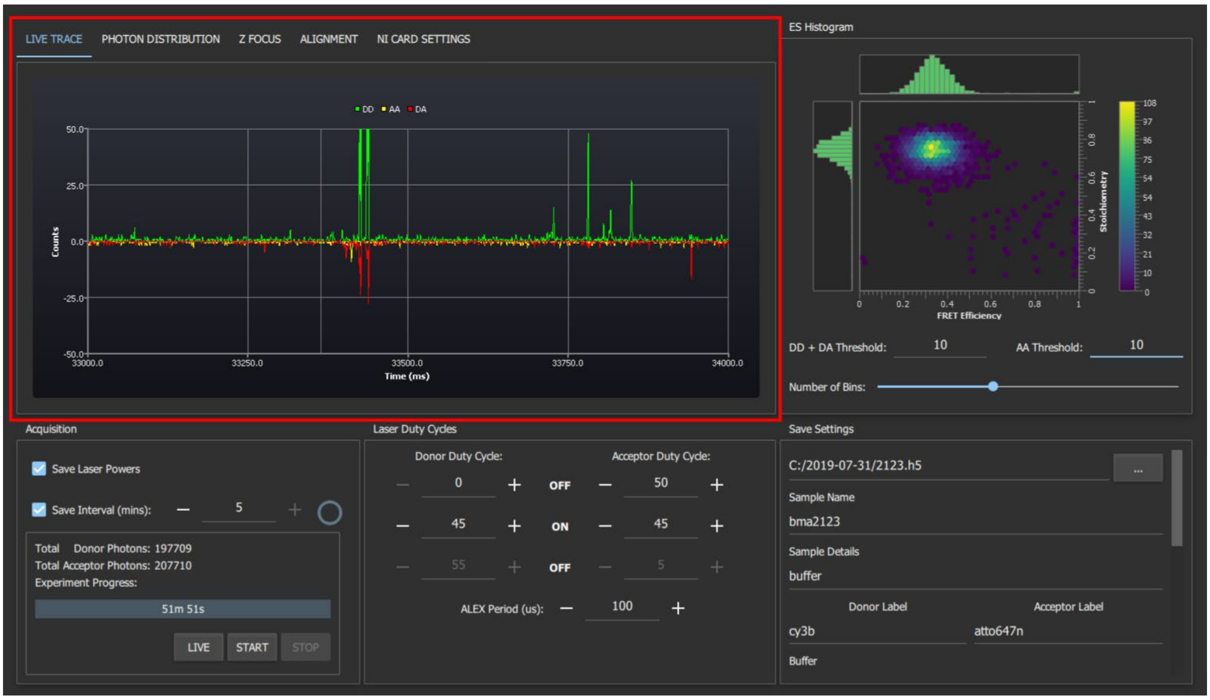

Supplementary Fig. N2.4: The live trace shows a real-time plot of the number of photons detected during live mode or an acquisition. The green trace corresponds to lasers detected on the donor APD when the donor laser is on, yellow corresponds to acceptor APD with acceptor laser and DA corresponds to acceptor APD with donor laser. The AA and DA traces are shown as inverted to make it easier to differentiate spikes in their traces from spikes in the DD trace.

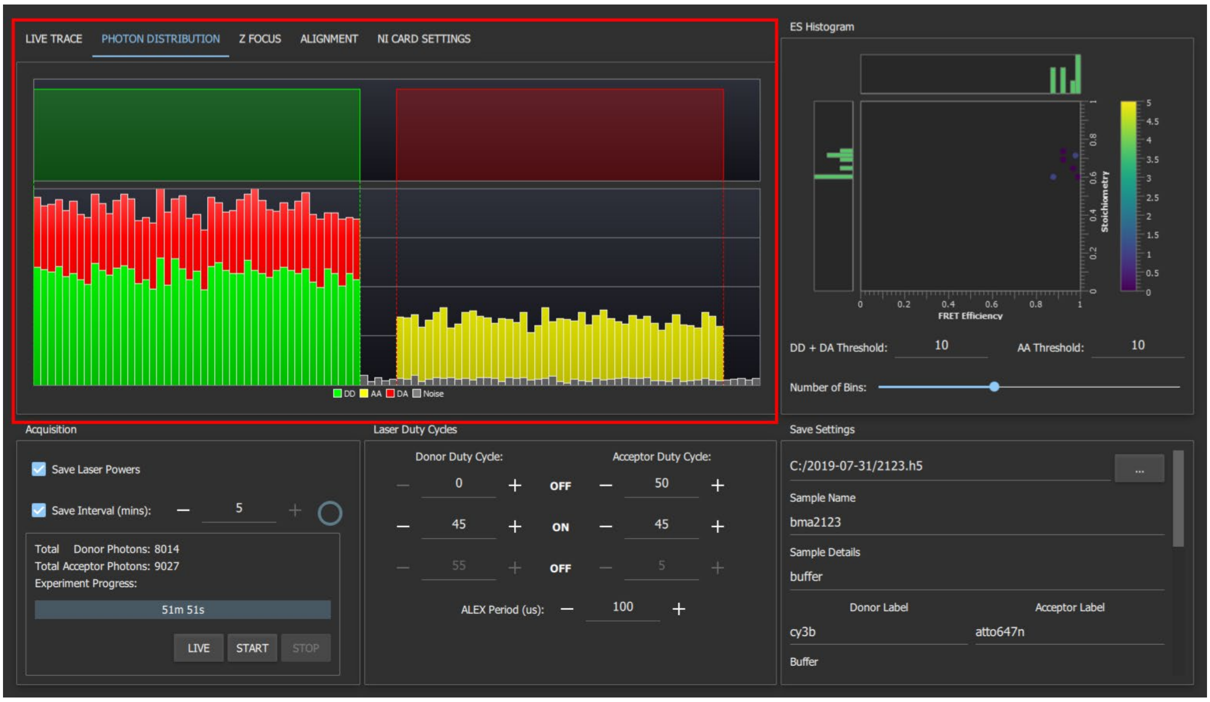

Supplementary Fig. N2.5: The photon distribution time tab shows a histogram of the photon arrival times with respect to the laser alternations (shown above the histogram). The red, green and yellow bars show the number of DA, DD and AA photons arriving at a given time relative to the laser alternation period whereas the grey bars show any detected photons that cannot be placed into one of these 3 categories (AD photons, or photons with no laser on). This panel can be a useful diagnostic to check the lasers are alternating correctly, especially if lasers with longer rise/fall times are used.

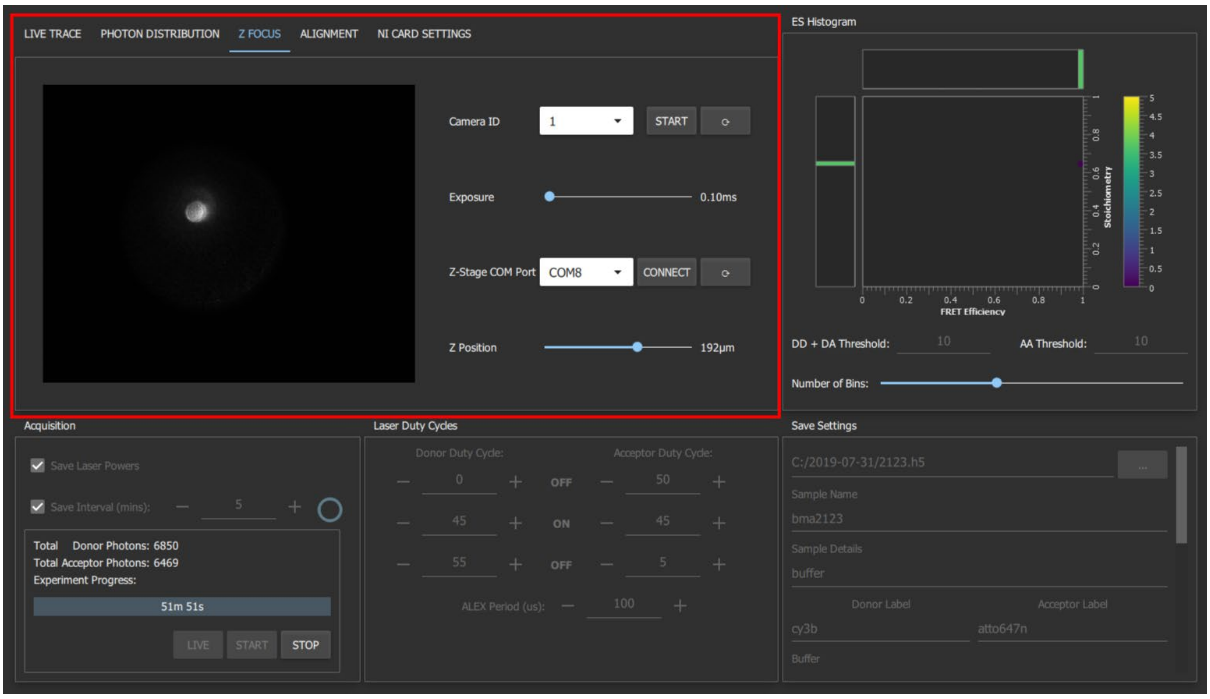

Supplementary Fig. N2.6: The Z-Focus tab allows you to control the z-position of the stage and shows the output of the CCD camera which is imaging the back-reflection of the sample. This allows you to focus the confocal spot into the sample. To operate, first place a drop of liquid on a coverslip on the objective. With either of the lasers on, move the slider for the z-position up until the back reflection shrinks to its smallest size. Then move slightly further up (~20 µm) so that the back reflection enlarges again. The confocal spot is now focused into the sample.

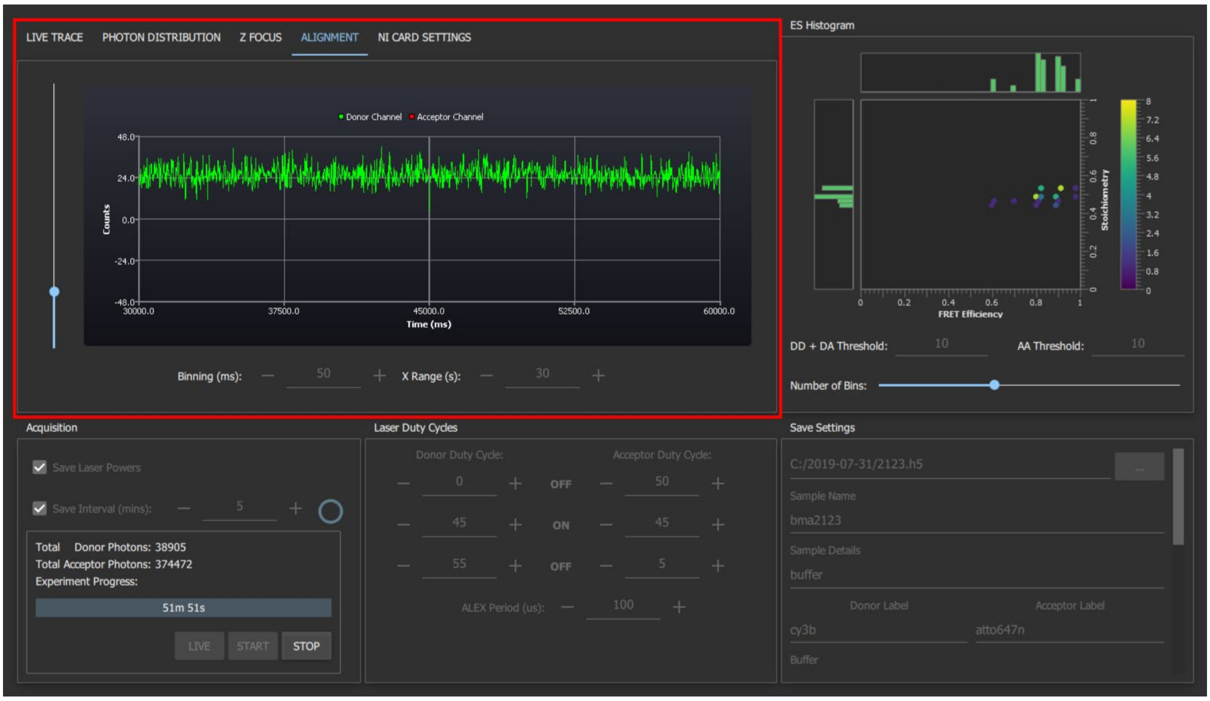

Supplementary Fig. N2.7: The alignment tab shows the number of photons detected on each detector, irrespective of which laser was on, and is able to plot a much higher flux of photons, which is useful for aligning components in the emission pathway. To make use of this, place a fluorescent sample on the coverslip which will emit in both channels when excited with the donor excitation laser (we use free Cy3B), then maximise the emission intensity reported by iteratively adjusting the positions of the APD lenses (L6 and L7), the pinhole, and if needed M3 and L4. Unlike the live trace graph, the binning and x-range can be adjusted. This allows you to select settings that result in a sufficiently smooth trace to allow you to easily align the system. For fine alignment it is recommended to use a high concentration so as to avoid fluctuations due to molecular diffusion, but low laser power such that the software can handle the signal from the APDs.

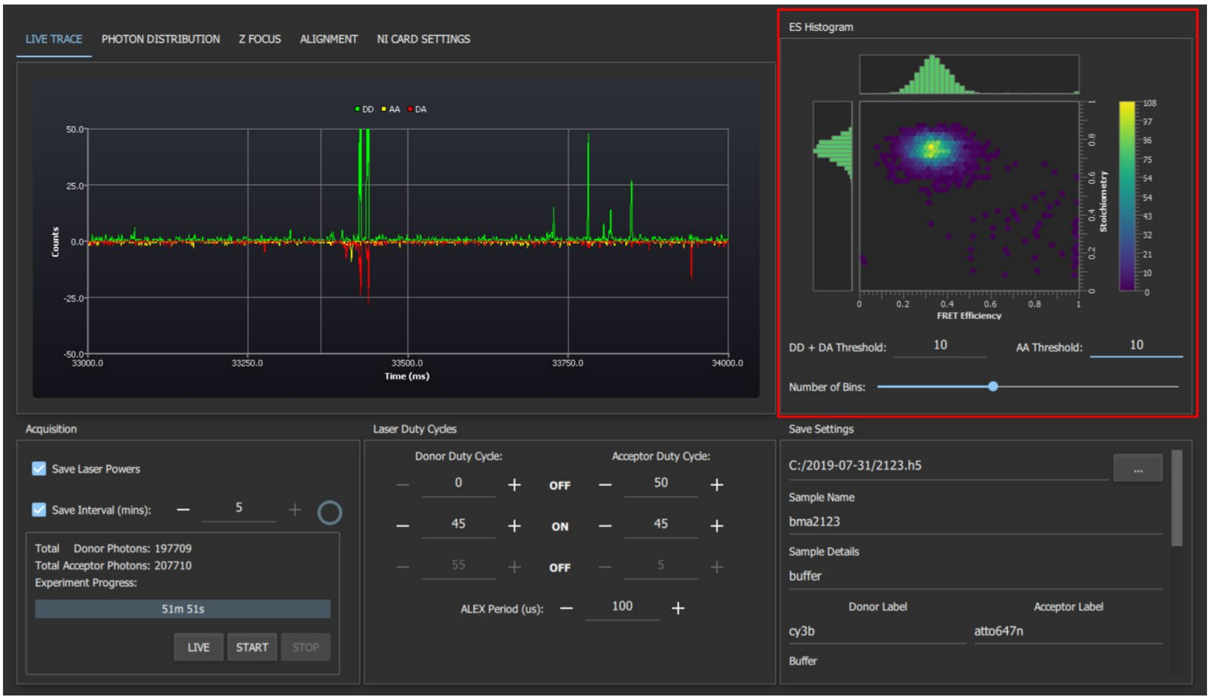

Supplementary Fig. N2.8: The live ES histogram shows an approximation of the hexagonal ES plots traditionally produced during ALEX-FRET analysis and helps give you a feel for any issues with your sample before you analyse the data. The FRET efficiency and stoichiometry are calculated and plotted for bursts which surpass both thresholds in a 1ms window. The number of bins slider can be adjusted to change the resolution of the histogram. Increasing it to the maximum value produces a plot that's more like a scatter graph if you prefer this to the hex plot.

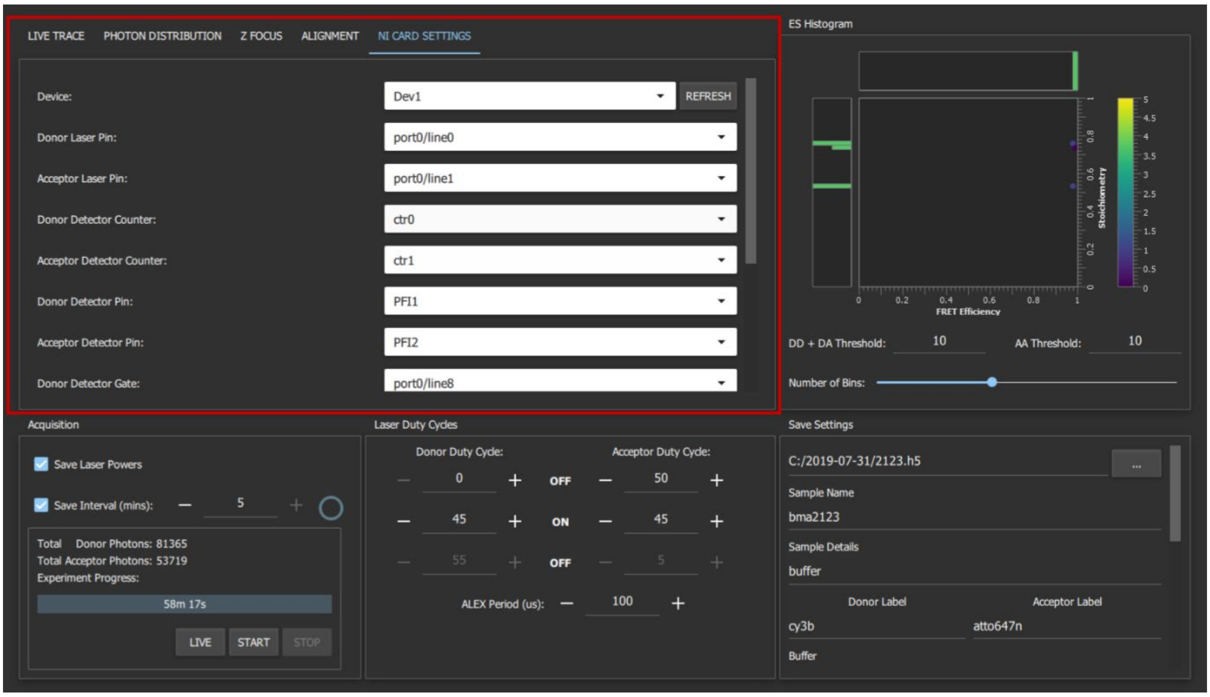

Supplementary Fig. N2.9: The NI Card Settings tab contains the advanced system settings that should not need to be changed unless you have constructed your system differently to our instructions. These settings are remembered between restarts.

Supplementary Note 3: *Operational Software - LABVIEW*

The LABVIEW implementation has three separate VI's for alignment, z-focussing, and acquisition, whereas smOTTER is capable of all and can save directly to HDF5 for quick analysis. However, the LABVIEW code is easily adapted by the community.

The pre-requirements for the LABVIEW VI's are:

[LabVIEW runtime 2016 64 bit](#)
[VISA runtime engine 16.0 64 bit](#)
[NI-DAQmx 16.0 64 bit](#)

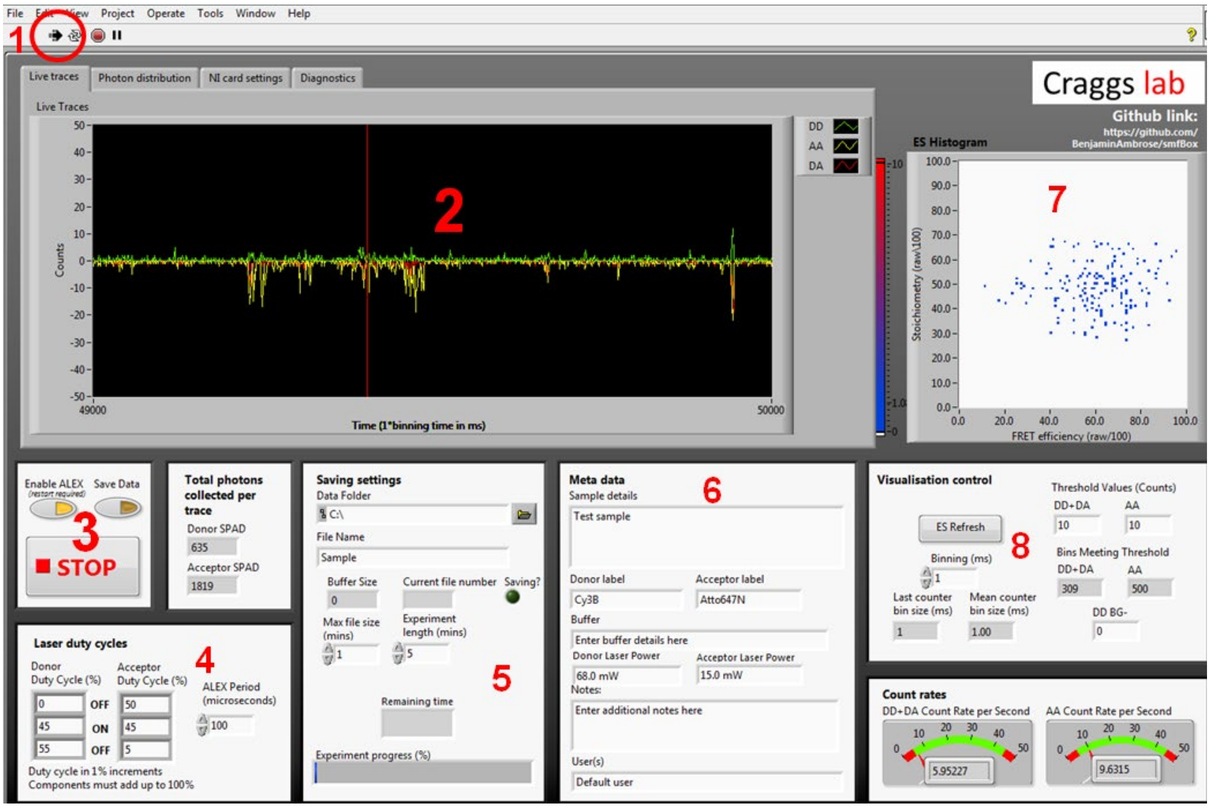

Supplementary Fig. N3.1: Front panel of the acquisition software. **1**. LABVIEW start button which begins ALEX and visualisation. **2.** Live time trace. Photons arriving at APD0 (donor) shown in green, and for ADP1 (acceptor) shown in red for photons under donor excitation and yellow for photons under acceptor excitation. **3**. Controls for ALEX, saving, and stopping. When you are ready to begin saving an acquisition, press the save data toggle during visualisation. **4.** ALEX controls. ALEX period controls the length of one full green-red alternation. The duty cycle boxes describe the amount of time each laser is off, then on, then off again for. This information is written into the metadata file, and pulled back out during file conversion to assign photons to channels. **5.** Location and name of saved files. You can set a max file size smaller than the experiment length and it will save as multiple files. **6.** Metadata typed here will be written into the meta data file, it will not affect the nature of the data itself. **7.** The visualisation control boxes affect the live 2D ES histogram on the bottom right. This histogram is built crudely and should not be used for rigorous data analysis, as it simply plots millisecond time bins exceeding the stated thresholds, rather than performing a full burst search. **8.** Controls for the live data visualisation. These do not affect the final saved data, only the live display.

The acquisition software controls the lasers and APDs and gives a live view of incoming smFRET data as well as creating the files needed for more in-depth analysis later. The first time you run the acquisition software you will need to go to the NI card settings tab and use the drop-down menus to select your NI card information, and the com ports each laser and APD are connected to.

Under normal operation; a user will typically place a sample on the scope, then press play (1) to check the quality/concentration before acquisition, we usually aim for 1-3 bursts per second to avoid co-incident events. If the sample needs to be changed again, press the stop button (3) before opening the lid of the smfBox to prevent ambient light from flooding the APD's. When happy with the sample, set the acquisition length and save location in (5), add metadata in (6), then press "Save Data" to begin the acquisition.

The diagnostics tab can be used to determine any problems with the software or hardware, and the photon distribution tab will give a live histogram of when photons have been detected with respect to the ALEX period. This can be a useful diagnostic of whether the lasers are being controlled properly without having to create a file for analysis first.

Supplementary Note 4: *Data Files*

The LABVIEW acquisition software saves raw txt files which contain the timestamp of each photon in one column and the detector it arrived at in a second column. For usage in analysis we have created python scripts which can convert these raw data to the open source photon-HDF5 format. This format contains not only the raw data but also can be written with sufficient metadata that the reader knows i. the origin and nature of the sample and experiment the data was acquired from and ii. Information required to interpret it. In this case the data originates from two unpolarised detectors acquired with a µs-ALEX scheme, and the script will write an HDF5 file which contains information for any analysis software to interpret it as such.

The scripts provided can convert a single TXT to a single HDF5, multiple TXT's to multiple HDF5's (batch conversion), or multiple TXT's for single HDF5's in the case of repeat acquisitions which you wish to analyse as a single dataset.

These conversion scripts are available as Jupyter Notebooks, as the analysis we provide is with the FRETBursts python module in Jupyter. However there is no reason why the code should not work in any other python environment as long as it has the phconvert module.

The standalone C++ software smOTTER will save directly to an HDF5.

Supplementary Note 5: *DNA sequences*

Name	Sequence
1a	5'- GAG CTG AAA GTG TCG AGT TTG TTT GAG TGT **T**TG TCT GG - 3'<br>3'- CTC GAC T**T**T CAC AGC TCA AAC AAA CTC ACA AAC AGA CC - 5' - biotin
1b	5'- GAG CTG AAA GTG TCG AGT TTG T**T**T GAG TGT TTG TCT GG - 3'<br>3'- CTC GAC T**T**T CAC AGC TCA AAC AAA CTC ACA AAC AGA CC - 5' - biotin
1c	5'- GAG CTG AAA GTG TCG AGT **T**TG TTT GAG TGT TTG TCT GG - 3'<br>3'- CTC GAC T**T**T CAC AGC TCA AAC AAA CTC ACA AAC AGA CC - 5' - biotin

Supplementary Table N5.1: Accurate FRET standard sequences used, identical to a previous study[1]. Highlighted T's in green and red represent C2 amino modified thymine residues labelled with Atto-550 and Atto-647N respectively. Acceptor strands are labelled with 5' biotins for surface immobilisation in the case of TIRF measurements in the original study (not used here).

Name	Sequence
MJW1	5' -T(C6 amino)-GGATTAAAAAAAAAAAAAAAAAAAAAAAAAAAAAAAAATCCAAAGGATGTATGGTAATGGGACGAAGAATGAGG 3'
MJW2	5' CCTCATTCTTCGTCCCATTACCA-T(C6 amino)-ACATCC 3'
MJW3	5' CCTCATTCTTCGTCCCAT-T(C6 amino)-ACCATACATCC 3'
MJW4	5' CCTCATTCTTCG-T(C6 amino)-CCCATTACCATACATCC 3'

Supplementary Table N5.2: DNA Hairpin Sequences. MJW1 was labelled with Cy3B, and MJW2, MJW3, MJW4 were labelled with Atto647N, then annealed to produce the high, mid, and low FRET hairpins respectively.

Supplementary Note 6: *Accurate FRET validation*

Double stranded DNA's labelled with Atto550 and Atto647n 23, 15, and 11-bp apart as described in Hellenkamp et al., (2018)[1] were diluted to approximately 0.1 nM in a buffer consisting of 5 mM Tris, pH 7.5, 20 mM $MgCl_2$ and 5 mM NaCl. 20 µl of diluted sample was placed on the smfBox on a coverslip passivated with BSA and covered with the cap of a micro centrifuge tube to prevent evaporation.

515 nm and 635 nm lasers were alternated with a 45/5/45/5 µs green/off/red/off cycle for two, half-hour acquisitions per sample. Time stamped data from the two APD's were converted to the open-source photon-HDF5 file format using custom Jupyter Notebooks (provided online[2]) using the phconvert python module.

Analysis was done using Jupyter Notebooks using the FRETBursts[3] python module (0.6.5), and Jupyter Notebooks (available in on the github[2]) following the method described in Hellenkamp et al., (2018)[1]. First "Correction Factor Finder Alpha-Delta.ipynb" is used followed by "Correction Factor Finder Gamma-Beta.ipynb" to determine correction factors, then "FRET Analysis.ipynb" is used to find the FRET efficiencies of each sample. The correction factors and corrected FRET efficiencies are given in Supplementary Table N6.1.

A brief description of the overall process is as follows: Background in each channel is estimated by means of an exponential fit of inter-photon delays greater than 1.5 ms, calculated for every 5 minutes of data. Bursts are identified using either an all photon sliding window algorithm, or dual channel burst search (DCBS) algorithm previously described[4] with L=10 and F=45 for both channels, and background is subtracted. For the spectral cross talk factors, data from all experiments are combined and an all photon search used, with only bursts with >50 photons in any channel selected, and ES plots produced (Supplementary Equations 1 and 2). Bursts with a stoichiometry >0.95 are selected as the donor only population and <0.175 for the acceptor only population. α and δ factors are calculated from E and S single Gaussian fits of donor only and acceptor only respectively using Supplementary Equations 3 and 4. A dual channel burst search (DCBS) is then used to extract doubly labelled bursts from each 30-minute acquisition, and bursts with >50 photons under green excitation used to find E and S with single Gaussian fits. 2D Gaussian positions of each 30-minute data set are then plotted together and fitted with Supplementary Equation 5 to obtain γ and β. 30-minute data sets are then combined for each sample, bursts extracted using DCBS with accurate FRET parameters applied, selected for >50 photons under green excitation, and FRET efficiency obtained via a single Gaussian fit.

	Hellenkamp et al., 2018	smfBox Data			Correction factors			
Sample	E	E	σ	N bursts	α	δ	γ	β
1a	0.15 ±0.02	0.17	0.07	694	0.081	0.076	0.856	0.848
1b	0.56 ±0.03	0.57	0.10	717				
1c	0.76 ±0.015	0.77	0.07	799				

Supplementary Table N6.1: Summary statistics for the accurate FRET validation. The means and standard deviations of FRET efficiencies from all participants in the benchmarking study[1] are shown, alongside the FRET efficiency mean (E) and width (σ) of a Gaussian fit to data from the smfBox, and the correction factors used (determined for all oligos).

Supplementary Note 7: *Asymmetric ALEX*

The rate and duty cycle of the green and red lasers of the smfBox can be completely customised using either acquisition software. It is even possible to leave one laser on all the time (for PAX, for example), or to leave one laser permanently off if ALEX is not required. For the experiments in this study we chose a duty cycle length of 100 µs, as this is fast enough to allow several observations with either laser per molecule (observation time of ~1 ms). If there are too few alternations then it reduces the ability of algorithms like the dual channel burst search to discriminate between molecules which have bleached during observation. However, the lasers have a rise/fall time of several microseconds (Supplementary N7.1), so repeating too fast would decrease the total time the lasers are on at full power. Furthermore, in either the case of converting to HDF5 from the LABVIEW software, or in directly saving to HDF5 in smOTTER, the details of the ALEX cycle are contained within the metadata, which is then read out by FRETBursts[3], so analysis is not further complicated by changing any of these parameters.

We have demonstrated the capabilities and effects of altering the ALEX cycle with a number of asymmetric excitation schemes, in which the amount of time either laser is on for is not equal. Typically ALEX schemes are symmetrical, however there is not strictly any reason why the red laser need be on for the same amount of time as the green. In fact, we argued that since all the information for the FRET efficiency comes under green excitation, it makes sense to maximise the time this laser is on. The four datasets shown (Supplementary Fig. N7.2) were taken using duplex 1b as described in the main text. A dual channel burst search (F = 15, m = 10) was used for analysis, and a burst selection of 50 photons under green excitation and 50 photons under red excitation.

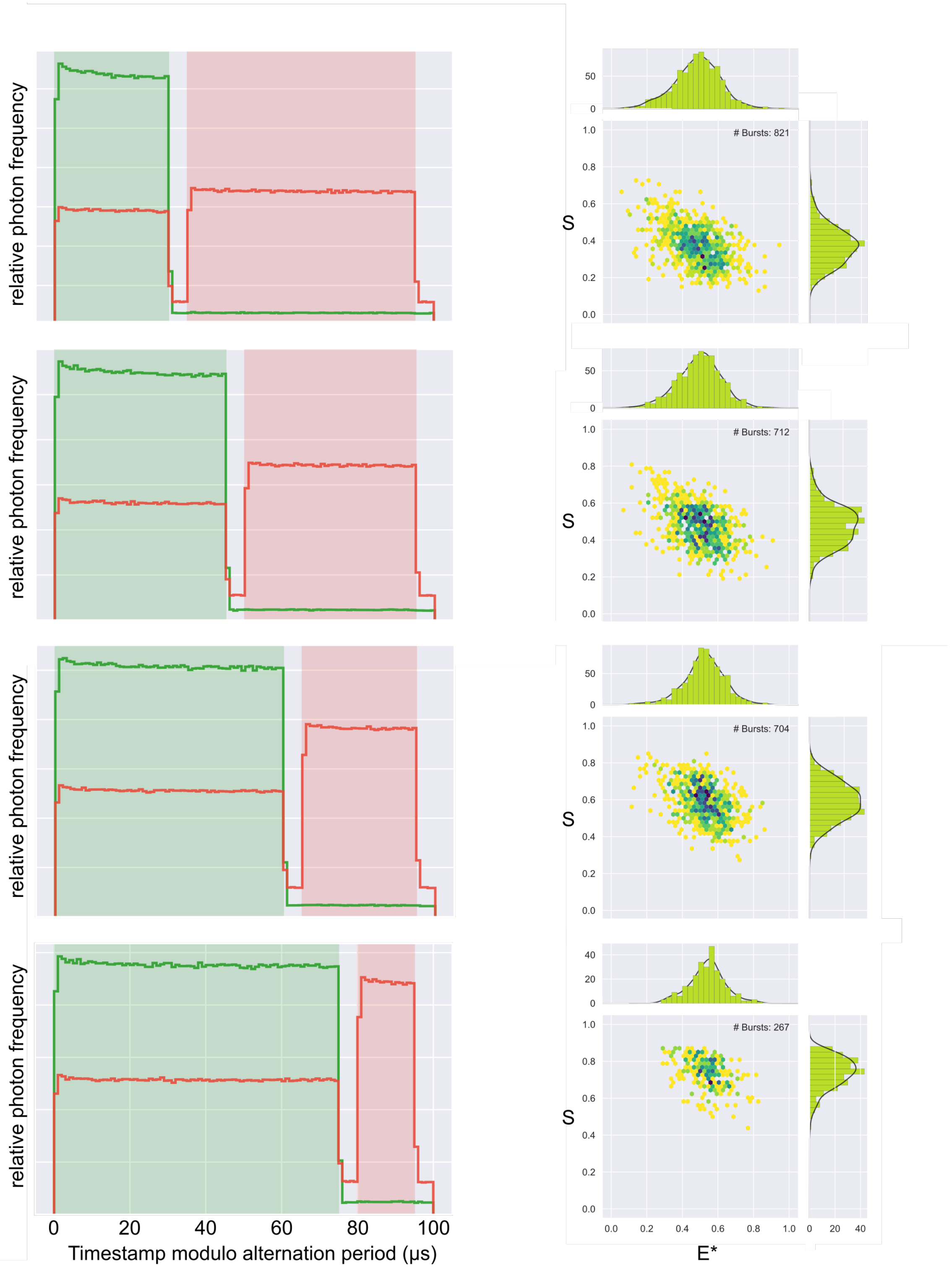

Supplementary Fig. N7.1: Four different ALEX schemes. On the left are photon arrival time plots with detected photons as green and red lines and shaded boxes representing which laser was on. On the right are uncorrected ES Histograms of DNA standard 1b measured with these ALEX schemes. From top to bottom the green/red excitation times are 30/60 µs, 45/45 µs, 60/30 µs, and 75/15 µs. In all cases a 5 µs wait time was left between each laser.

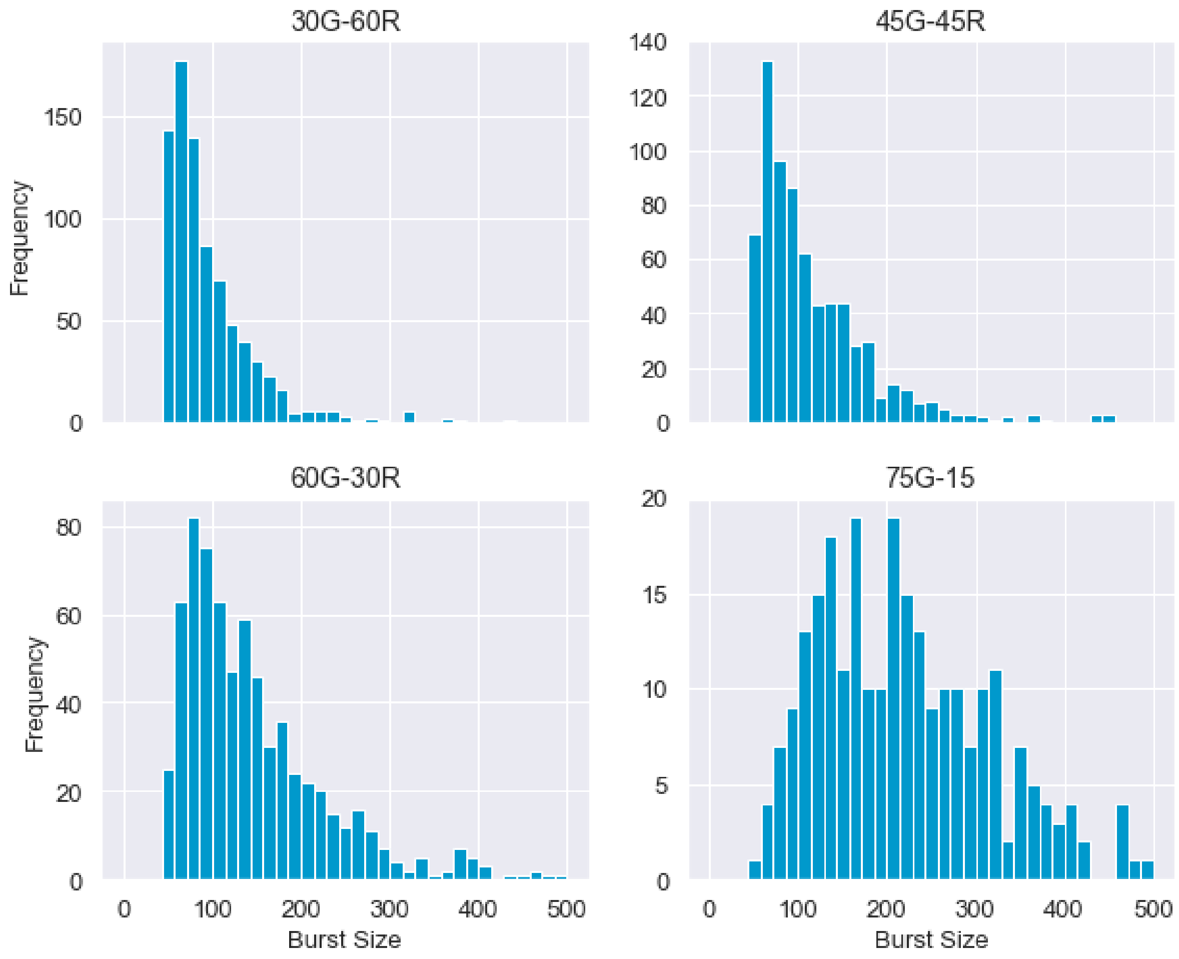

Supplementary Fig. N7.2: Burst size histograms for each excitation scheme. Plotted are the number of photons under green excitation after the selection of >50 photons, which is why there are no bursts <50 photons shown.

Changing the relative amounts of excitation by either laser shows a predictable trend (Supplementary Figure N7.1). The S position in each ES plot increases with the length of green excitation, due to the increased number of photons detected under green excitation. However, two other effects become apparent. Firstly, the width of the distribution in E decreases due to the decreased contribution of shot-noise from the increased number of photons acquired. Secondly, the number of doubly labelled bursts which are found by the burst search algorithm decreases due to the reduced information under acceptor excitation, which is required to confidently assert that any burst is doubly labelled. From these two effects it is easy to see that there may be an optimum excitation scheme, which may not necessarily be symmetrical, since the quantity of information obtained under donor excitation is greater than that obtained under acceptor excitation. As expected, we can see that the burst size histogram shifts to increasing number of photons under green excitation when the green excitation period is increased (Supplementary Figure N7.2), consistent with the reduction in width in FRET efficiency histogram due to decreased shot noise. Whilst the width in a FRET efficiency histogram is dependent on the mean FRET efficiency, we would expect this effect to still hold at any given FRET efficiency, however the decrease in width would be proportional to the original width at that FRET efficiency.

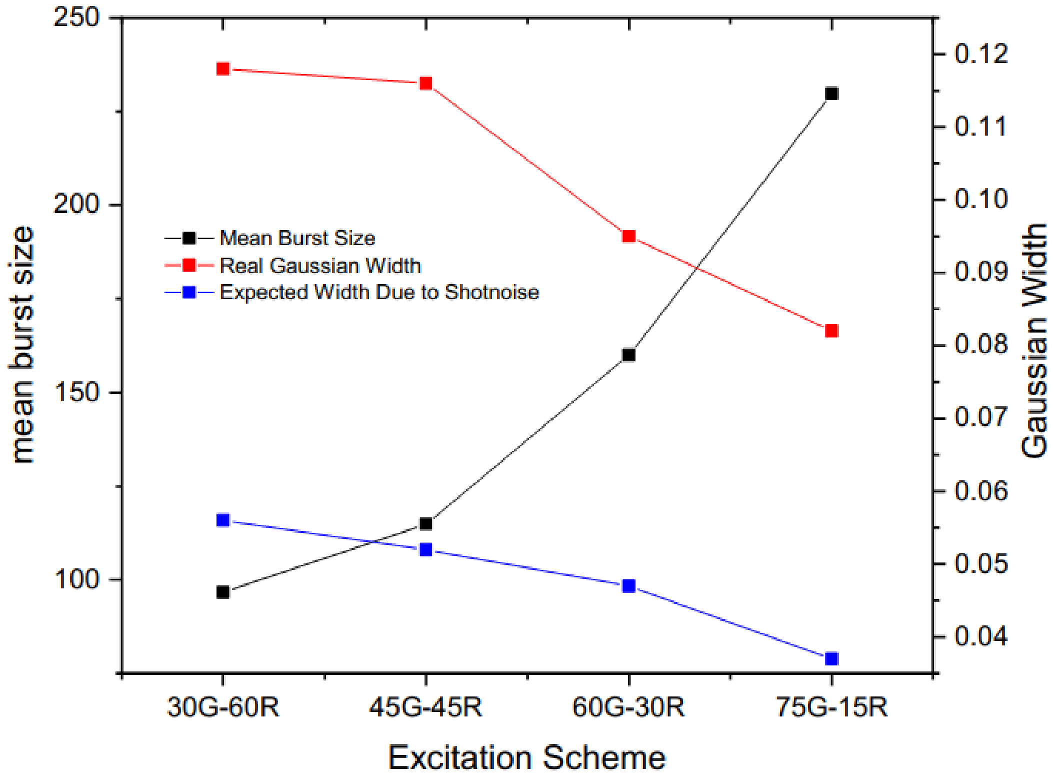

Supplementary Fig. N7.3. Burst statistics under each excitation scheme, showing: the mean number of photons under green excitation (black); the width of the Gaussian fitted to the 1D FRET efficiency distribution (red); and the expected Gaussian width computed from the burst size distribution (blue).

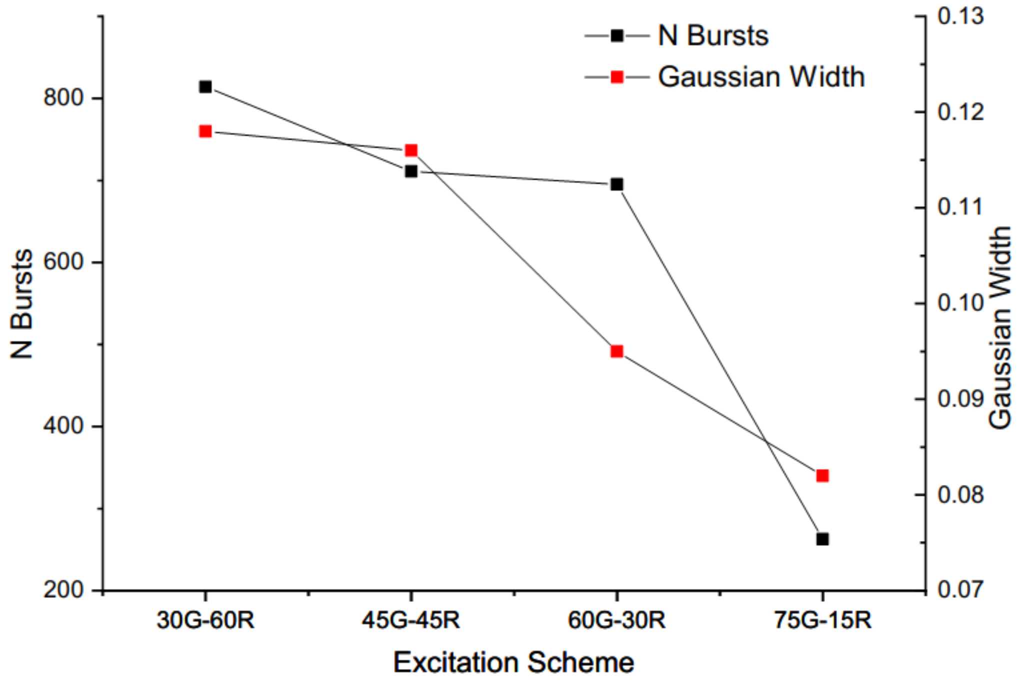

Supplementary Fig. N7.4: Four different excitation schemes with the number of bursts found by a DCBS and the width of the Gaussian distributions fitted to the FRET efficiencies.

To further emphasise this point, we see that Gaussian width decreases with increasing burst size, as can be predicted by a binomial computation of shot noise from the burst size distribution (Supplementary Figure N7.3). Note that the width of the Gaussian distribution fitted to the experimental data is larger than predicted from shot noise alone, as shot noise is not the only form of noise present, but represents a considerable proportion, nonetheless. Other forms of noise which make up for the discrepancy are background contribution, and flexing of the DNA molecule, which has been previously discussed in detail[4–6]. Finally, in Supplementary Figure N7.4 we can see the effects of each excitation scheme on both Gaussian width and number of detected bursts. Whilst more testing may be necessary to fully explore this relationship, it can be seen phenomenologically that increasing donor excitation to twice that of the acceptor can markedly decrease the width of the obtained FRET distribution without having to resort to increasing laser power (and hence increasing photobleaching), whilst having little to no effect on the ability to confidently assert that a burst is doubly labelled. From this plot we would recommend as an immediate improvement over symmetric ALEX a ‘sweet-spot’ of 60G-30R for the duty cycle, to both maximise the number of detected bursts and minimise the shot noise. The exact sweet spot may vary depending on application however; if trying to detect a relatively small shift in FRET efficiency then tighter histograms at the expense of a few less bursts as shown may be desirable, however if quantifying two populations that are already well separated in FRET efficiency, then tighter histograms are less of an advantage. With a large parameter space (laser power, photobleaching, oxygen scavengers, burst length, burst size, noise, etc.) it is difficult to prescribe an objectively optimum asymmetric-ALEX ratio for any given application. However, the ability to alter the duty cycle available in the software provided allows the user to optimise as they see fit.

Supplementary Note 8: *Verification of Dynamics*

Whilst dynamic Photon Distribution analysis (dPDA) is capable of recovering the rates of interconversion between two different FRET species, by analysing FRET efficiency histograms, other methods provide more powerful tests for the existence of dynamics in a system. If fluorescence lifetimes are available, then an E-tau plot can discriminate dynamic and static bursts via deviation from a "static FRET line"[5]. If, as in our experiments, only intensities are available, then either the burst variance analysis (BVA) method[7] or two channel kernel density estimator[8] (2CDE) can be used to test for dynamics. Both methods use the photon arrival times within each burst to "score" for dynamics, BVA uses the variance of FRET efficiencies of subdivided bins within each burst, whereas 2CDE compares kernel density estimators between channels. As each burst is scored for discrimination between static/dynamic, these methods can be used as hypothesis testing to verify that dynamic populations are present before being quantified by dPDA. Here we show the FRET-2CDE method as implemented using PAM[9] applied to the hairpins in this study, using static dsDNAs as a control to demonstrate not only supporting evidence that the hairpins are dynamic, but that this form of analysis is also possible with the smfBox platform (Supplementary Fig. N8.1).

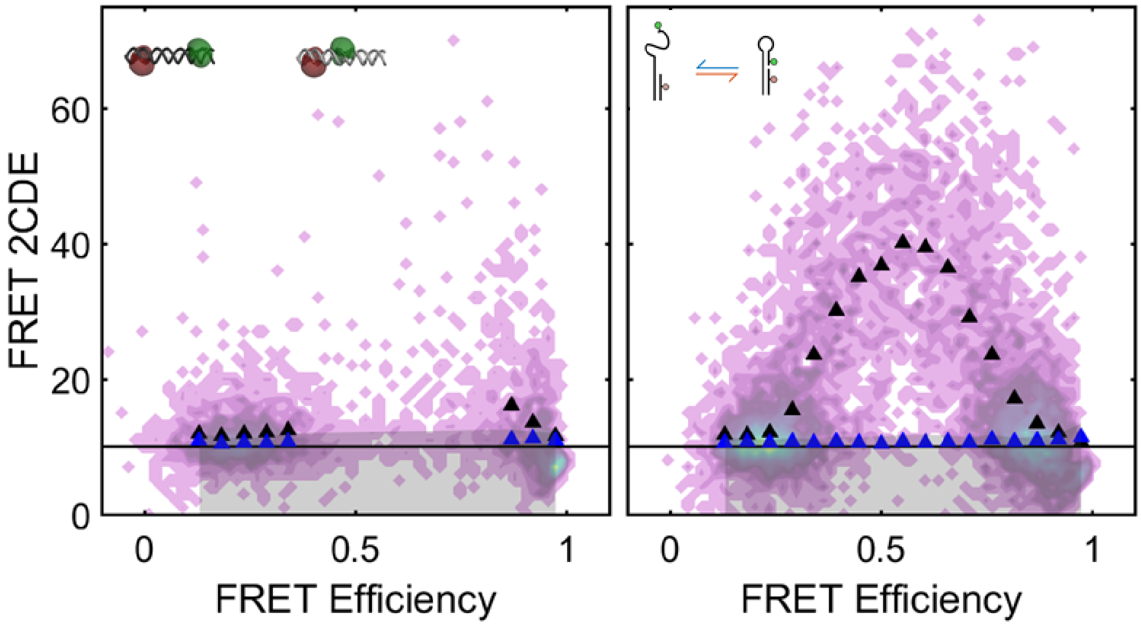

Supplementary Fig. N8.1: 2CDE plots of a static dsDNA mixture (left) and a dynamic hairpin (right). FRET-2CDE analysis[8] can be done in PAM on data from the smfBox in order to verify the presence of dynamics in the sample. A high 2CDE value (average shown in black triangles) suggests that bursts are dynamic, i.e. Interconverting between FRET efficiencies during observation, whereas bursts with low 2CDE values fall along a straight line (blue triangles) suggesting a static FRET efficiency.

Supplementary Note 9: *Possible Expansions*

Due to the modular nature of the smfBox it can be readily expanded for use in other confocal based techniques beyond the scope of this paper. Various possible additions (example suppliers) and the enabled modalities include:

- 2 axis scanning stage (Piezoconcept / MadCity Labs / PI) to enable imaging by sample scanning.
- Pulsed lasers and controllers (Picoquant) to enable PIE and picosecond resolution time-correlated single-photon counting (TCSPC) electronics (Picoquant) to enable pulsed-interleaved excitation experiments[10].
- Additional APDs (Excelitas) and polarisation optics (Thorlabs) to enable multiparameter fluorescence detection experiments[11], and nsFCS[12].
- An objective and stage heater (Okolab) to maintain the sample at a physiologically relevant temperature, or a range of temperatures for thermodynamic characterisation.

In addition to small organic fluorophores, we expect the smfBox to be capable of detecting fluorescent-protein-labelled biomolecules, as we have shown in the past on similar instruments[13]. To this end, we have created an FPbase profile for the smfBox (https://www.fpbase.org/microscope/Aa5VpxP9ff3nmsdjnYBRUb/) , which allows users to check the compatibility of fluorescent proteins (and other fluorophores) with our instrument, or their version of it (with customised optics / lasers). However, we note that while there are many green/yellow fluorescent proteins which would be detectable in the donor channel with similar efficiency as our organic fluorophores, e.g. mVenus, there are fewer suitably bright fluorescent proteins in the far-red range which can be excited by the 638 laser. Use of two-colour FRET with fluorescent proteins would therefore require a reconfiguration of the lasers and filters, for example a 488/568 combination would likely work well the wealth of green/red FP's (ie. mNeonGreen/mScarlet), which we estimate to have comparable brightness to the organic fluorophores we typically use in smFRET.

Supplementary Note 10: *FCS, FCCS*

The smfBox as presented is capable of FCS and FCCS with no hardware modifications. Here we show example data (Fig N8.2) using a free dye of known diffusion coefficient (Rhodamine 6G) to determine the confocal volume, and a 37bp doubly labelled duplex DNA which is determined to have a diffusion coefficient of 88 $\mu m^2 s^{-1}$, consistent with expected literature values.

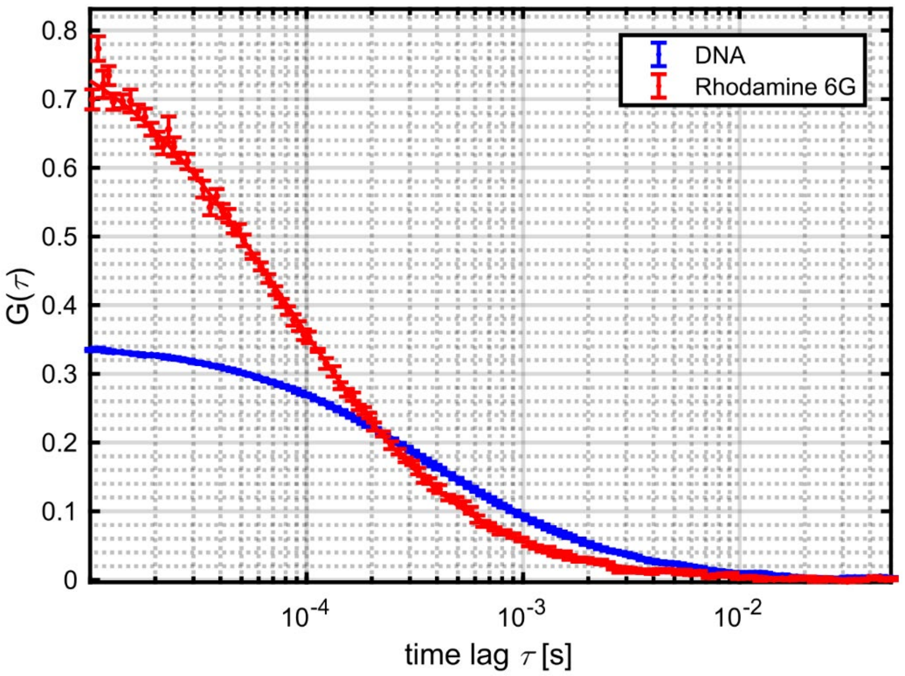

Supplementary Fig. N10.1: Correlation curves for Rhodamine 6G and a 37 bp doubly labelled DNA.

Samples were diluted to a concentration higher than would be normally used for single-molecule experiments (~ 1 nM), in water in the case of Rhodamine 6G, and in buffer (5 mM Tris, pH 7.5, 20 mM $MgCl_2$ and 5 mM NaCl) in the case of the doubly labelled DNA. Data were acquired for 3 minutes under continuous wave excitation with the 515 nm laser, at a reduced laser power (100 uW before the objective). Cross correlation curves (DD x DA) were generated and fitted using PAM[9]. A simple 3D diffusion model was used, with the diffusion coefficient for Rhodamine 6G being fixed at 414 $\mu m^2 s^{-1}$ (from ref. [14]) and the confocal volume parameters were shared between both curves.

Sample	Counts (KHz)	Brightness (KHz)	N[a]	D ($\mu m^2 s^{-1}$)[b]	W_0 (µm)[c]	Z_0 (µm)[d]
R6G	58	135	0.43	414	0.36	4.31
DNA	205	199	1.03	88	0.36	4.31

Supplementary Table N10.1: FCS curve fit parameters. The value of 88 $\mu m^2 s^{-1}$ determined for the diffusion coefficient of the 37 bp doubly labelled DNA is typical for an oligomer of this length[15]. Note that as we are underfilling the back aperture of the objective, the dimensions of the confocal volume are, as expected, greater than that of a diffraction limited spot. **a**: average number of molecules in the confocal volume **b**: diffusion coefficient (fixed for Rhodamine 6G, measured for DNA) **c, d**: lateral and axial dimensions of the confocal volume, respectively, calculated by fitting the R6G data assuming a 3D-Gaussian profile.

Supplementary Figures

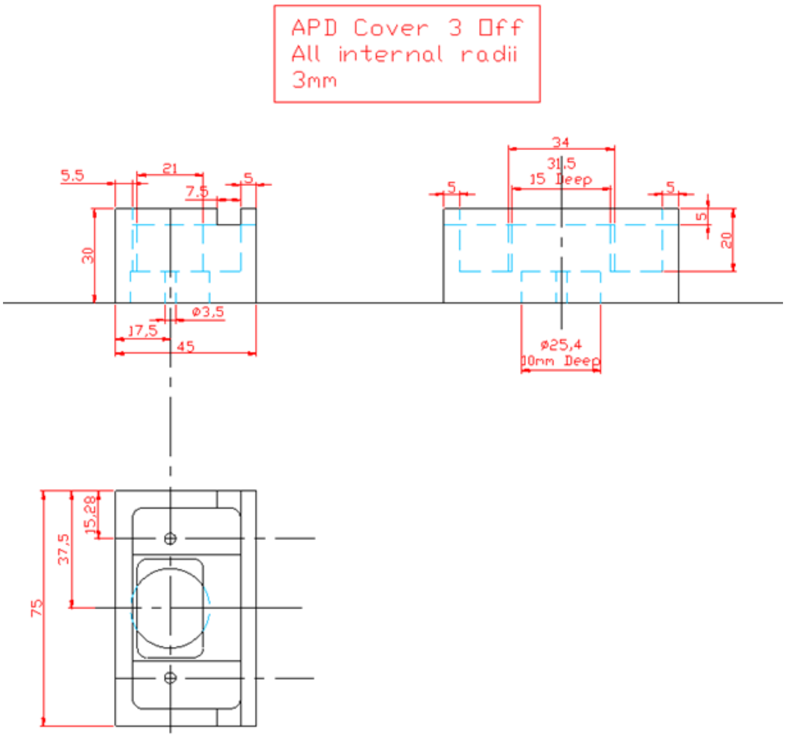

Figure S1: APD cover. All dimensions given in millimetres.

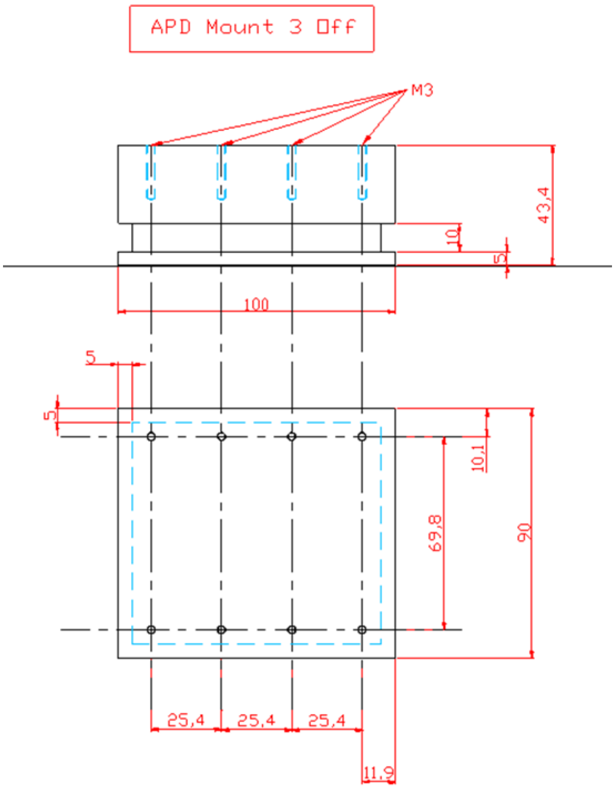

Figure S2: APD Mount. All dimensions given in millimetres.

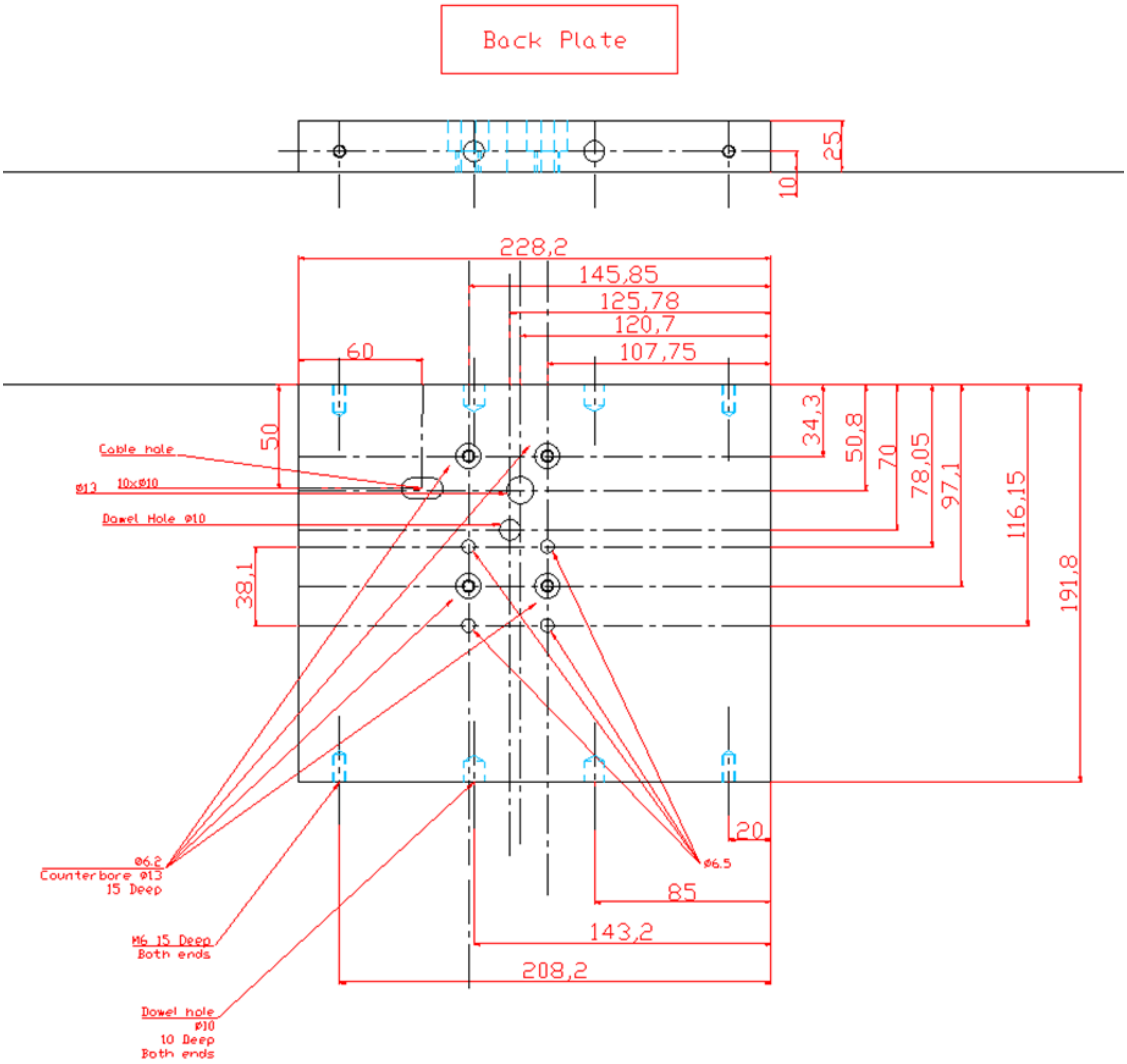

Figure S3: Back plate. All dimensions given in millimetres.

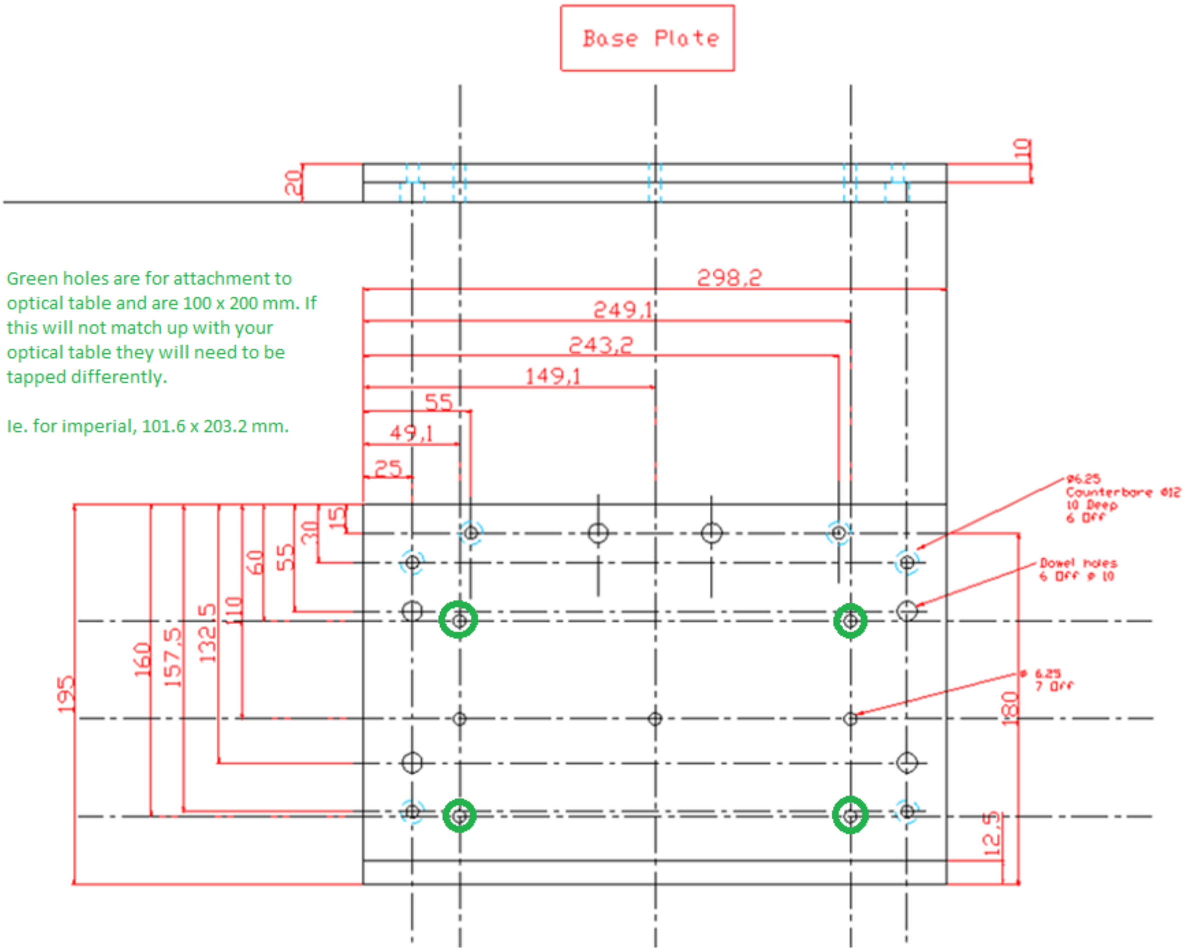

Figure S4: Base Plate. All dimensions given in millimetres.

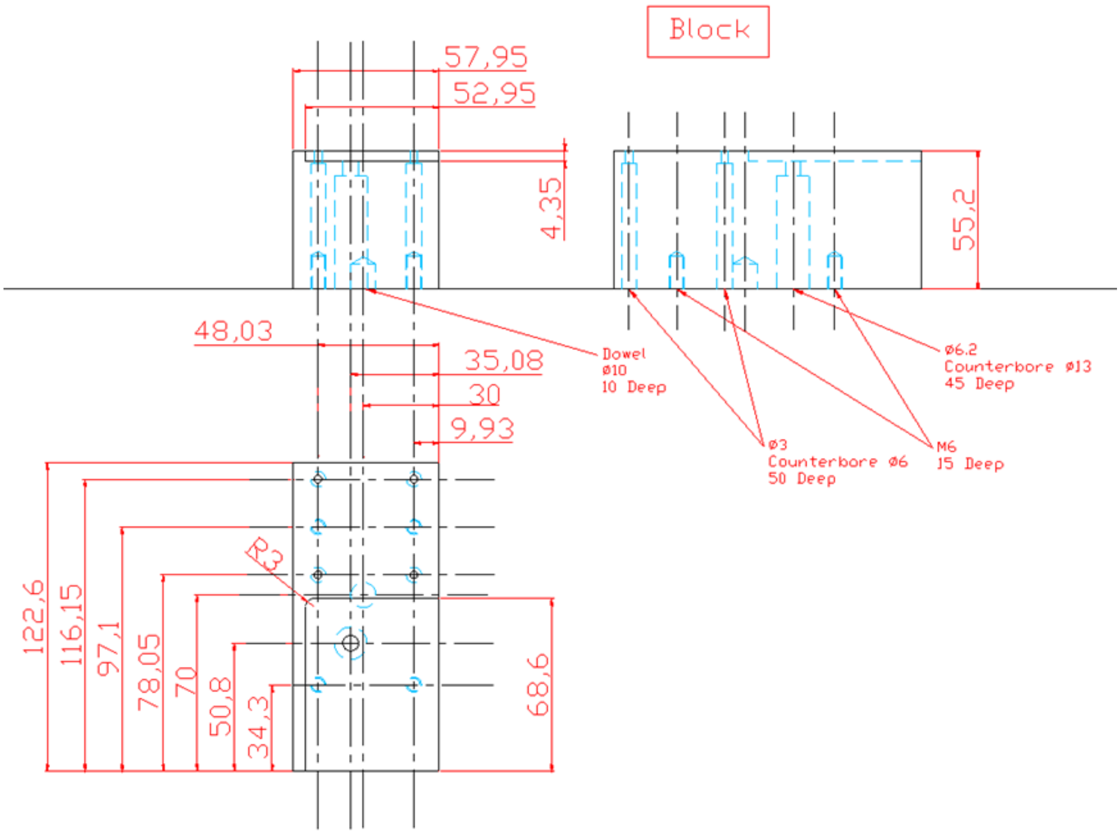

Figure S5: Block. All dimensions given in millimetres.

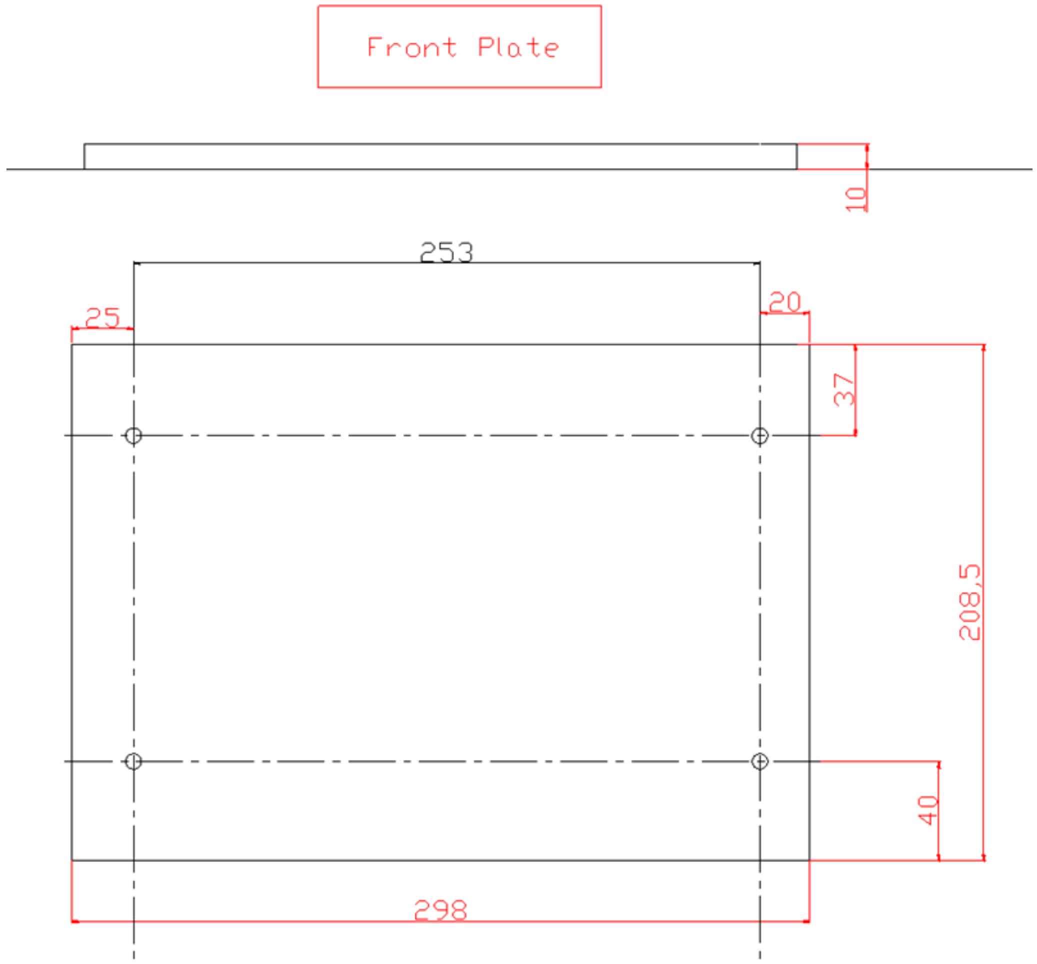

Figure S6: Front plate. All dimensions given in millimetres.

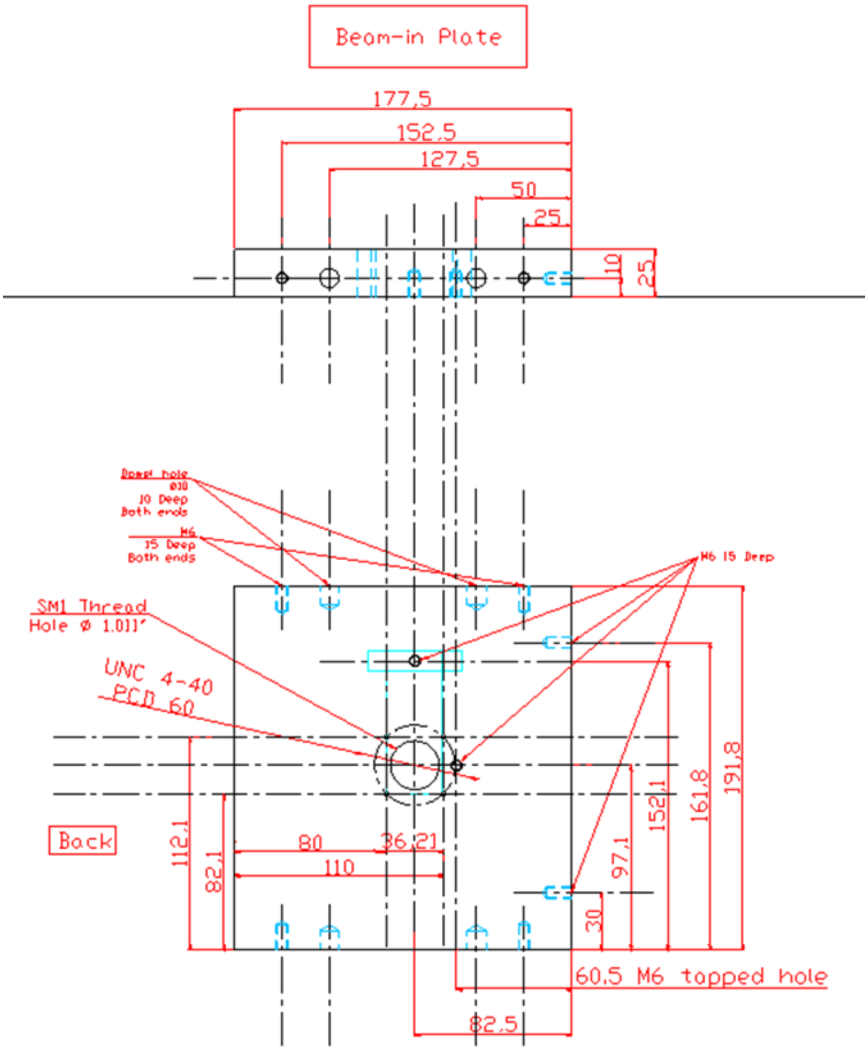

Figure S7: Laser-in plate. All dimensions given in millimetres.

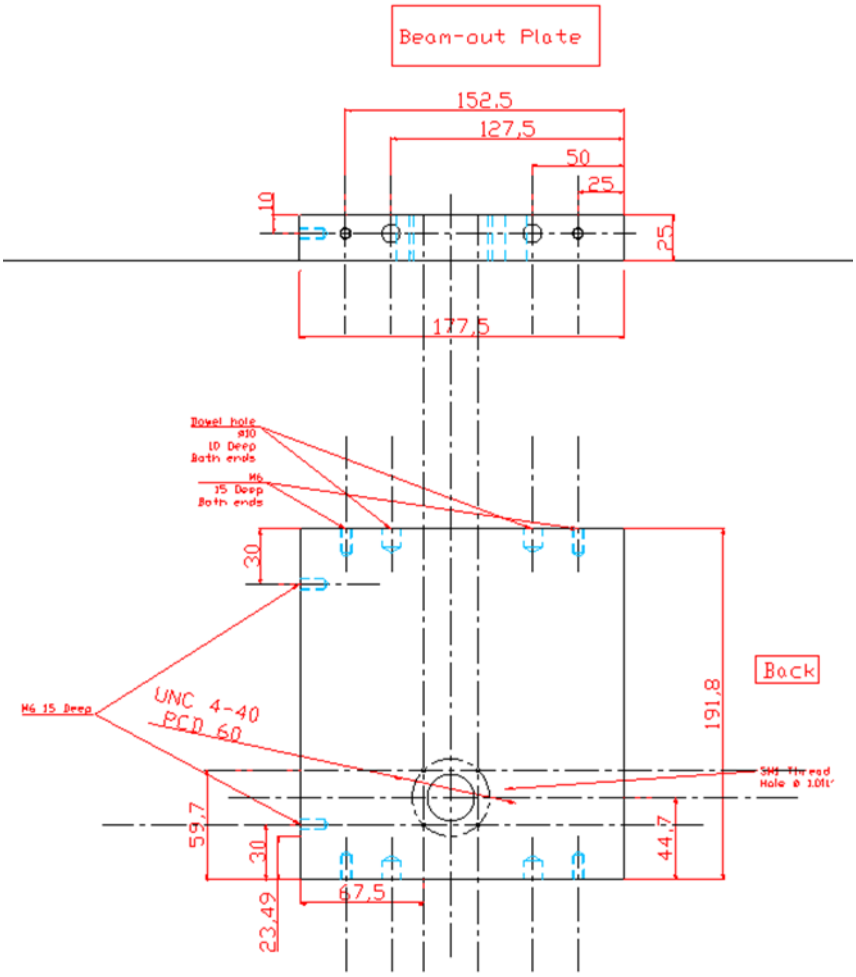

Figure S8: Laser-out plate. All dimensions given in millimetres.

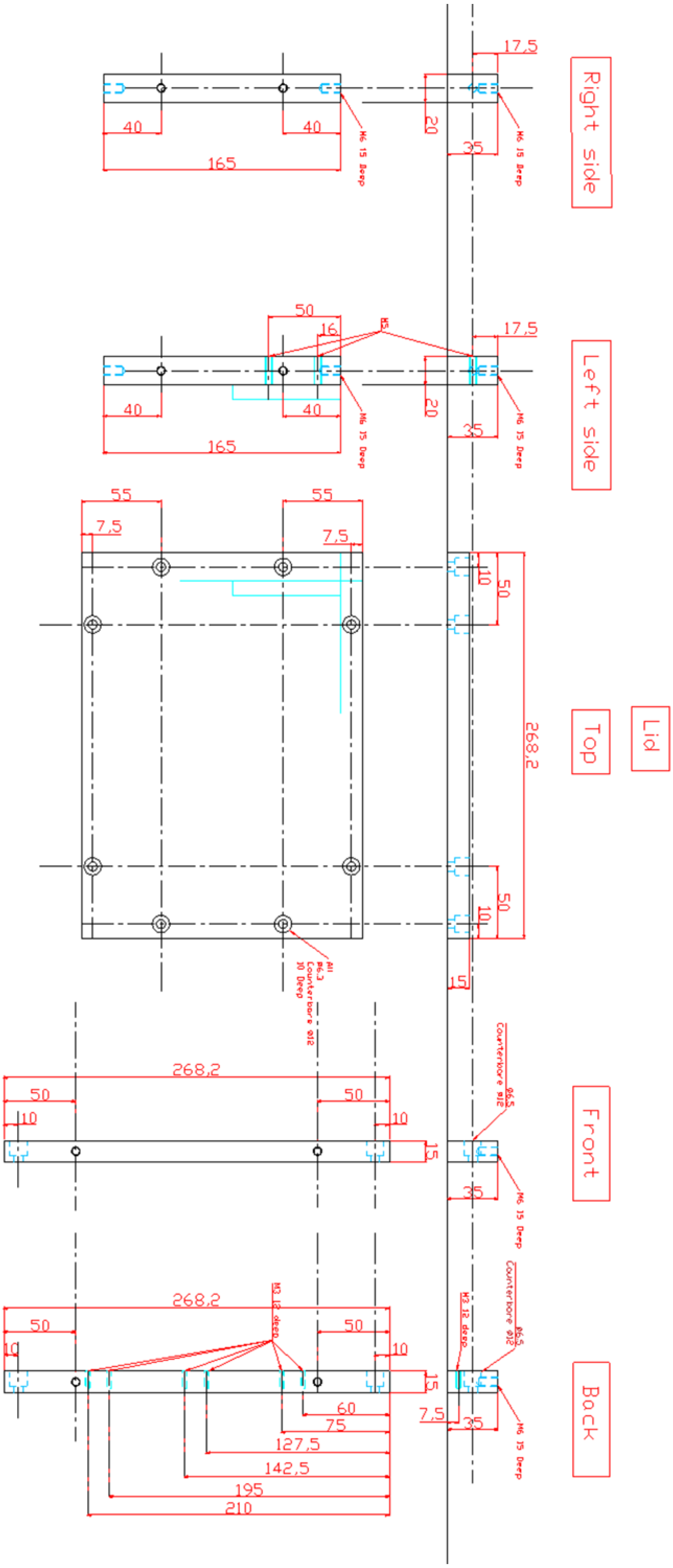

Figure S9: Lid. All dimensions given in millimetres.

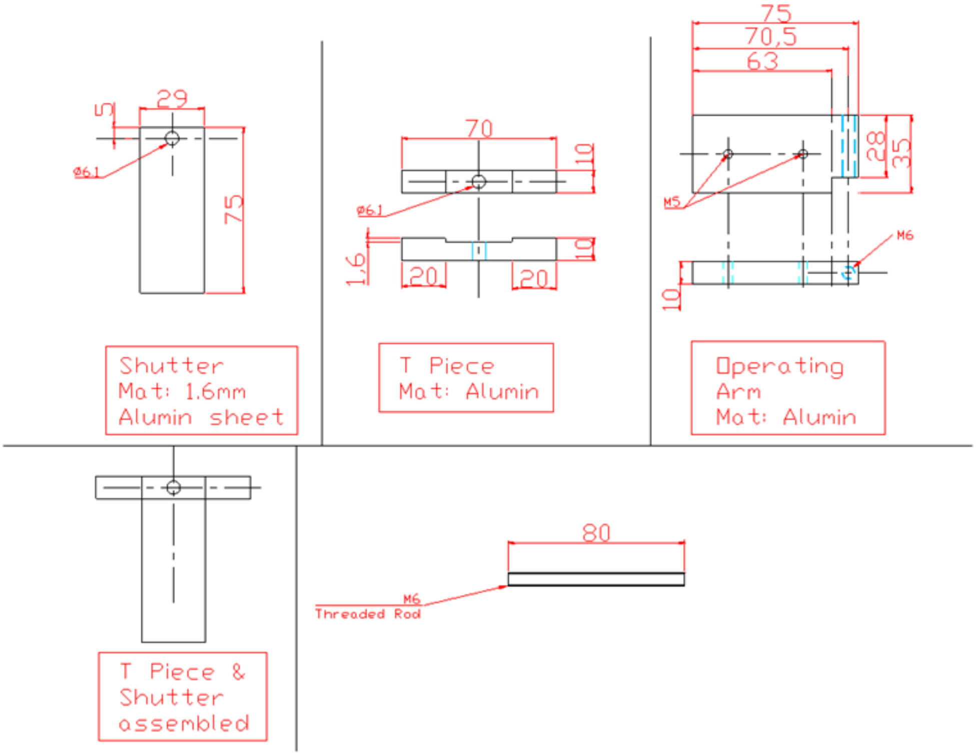

Figure S10: Shutter. All dimensions given in millimetres.

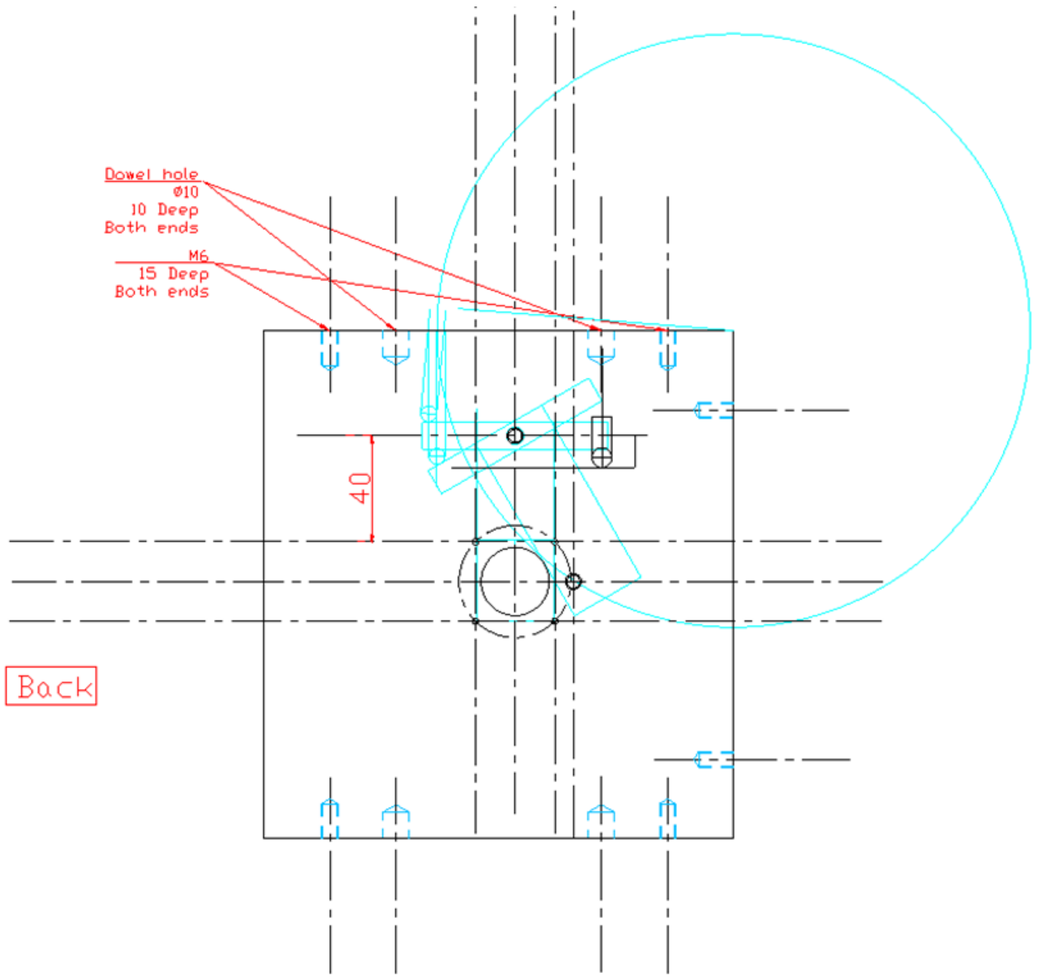

Figure S11: Shutter assembly. All dimensions given in millimetres.

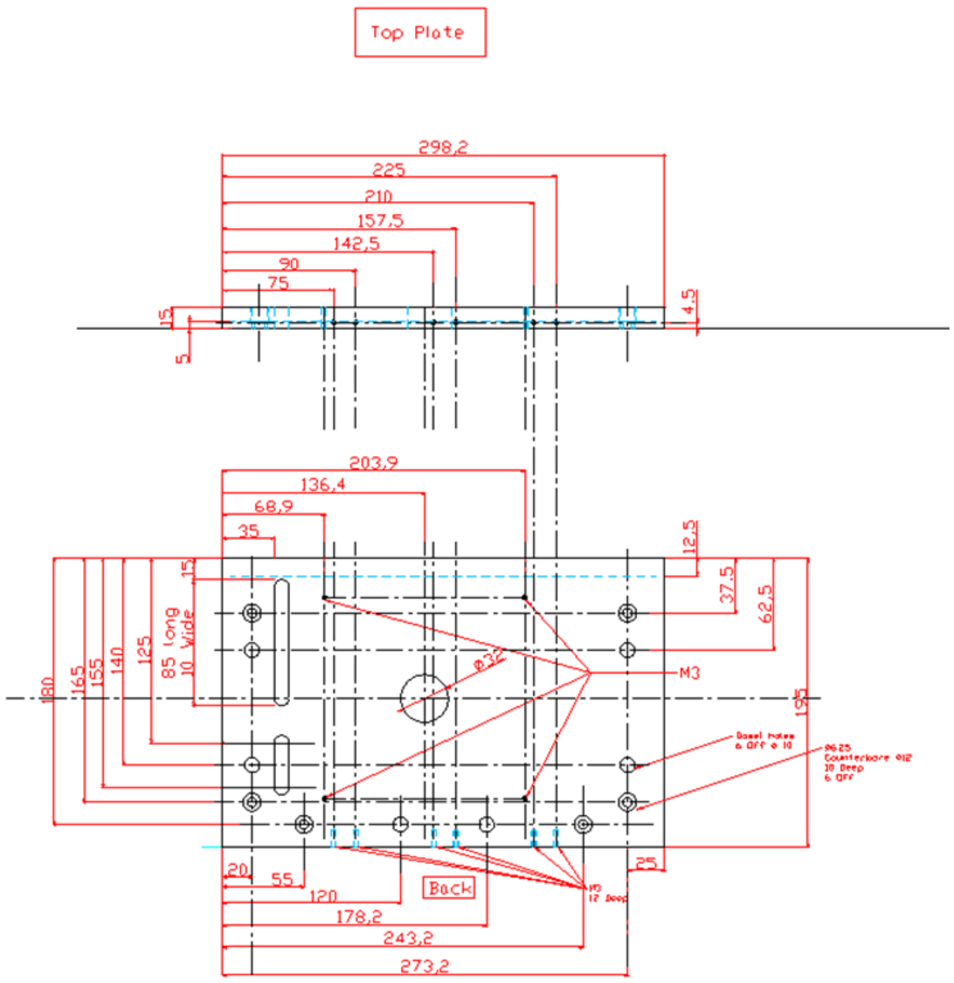

Figure S12: Top plate. All dimensions given in millimetres.

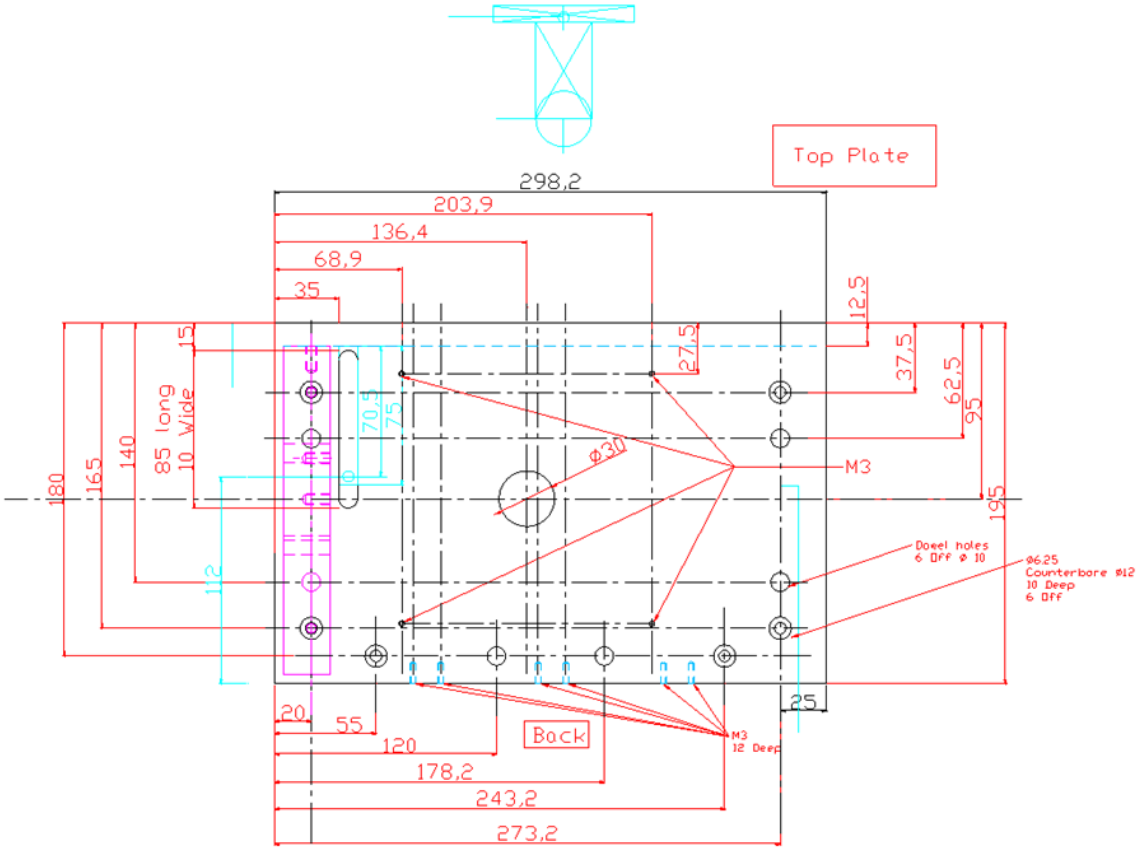

Figure S13: Top plate assembly. All dimensions given in millimetres.

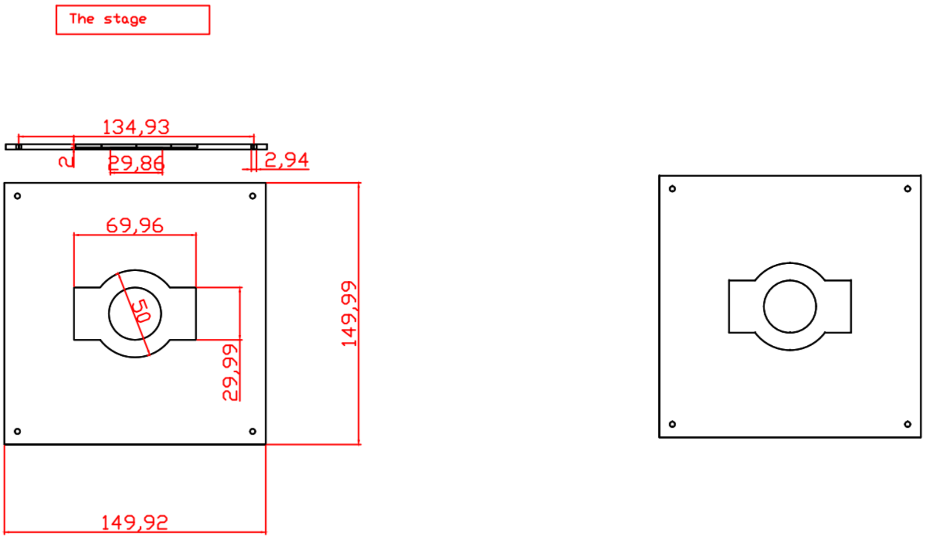

Figure S14: Sample Stage. All dimensions given in millimetres.

Supplementary Equations

$$\text{Proximity Ratio, } PR = E^* = \frac{DA}{DD+DA} \tag{1}$$

$$S = \frac{DD+DA}{DD+DA+AA} \tag{2}$$

$$\alpha = \frac{E_{\text{app}}^{\text{D}_{\text{only}}}}{1- E_{\text{app}}^{\text{D}_{\text{only}}}} \tag{3}$$

$$\delta = \frac{S_{\text{app}}^{\text{A}_{\text{only}}}}{1- S_{\text{app}}^{\text{A}_{\text{only}}}} \tag{4}$$

$$S_{\text{app}}^{DA} = \frac{1}{(1+ \gamma\beta + (1-\gamma)\beta E_{\text{app}}^{DA})} \tag{5}$$

Where:

DD indicates the sum of the raw photon counts on the donor APD under donor excitation, *DA* indicates the sum of the raw photon counts on the acceptor APD under donor excitation, and *AA* indicates the sum of the raw photon counts on the acceptor APD under acceptor excitation.

$E_{\text{app}}^{\text{D}_{\text{only}}}$ represents the mean of a Gaussian fit to the FRET efficiency of donor only species in the background corrected histogram, used to obtain the leakage parameter, α.

$S_{\text{app}}^{\text{A}_{\text{only}}}$ represents the mean of a Gussian fit to the stoichiometry values of donor only species in the background corrected histogram, used to obtain the direct excitation parameter, δ.

E_{app}^{DA} and S_{app}^{DA} represent the background and cross-talk-corrected FRET efficiency and stoichiometry, respectively, of individual bursts used to obtain the γ and β correction parameters.

Supplementary References

1. Hellenkamp, B. *et al.* Precision and accuracy of single-molecule FRET measurements—a multi-laboratory benchmark study. *Nat. Methods* **15**, 669–676 (2018).
2. Ambrose, B., Steele, E.M. smfBox - Craggs Lab Github. https://craggslab.github.io/smfBox/. (2019)
3. Ingargiola, A., Lerner, E., Chung, S., Weiss, S. & Michalet, X. FRETBursts: An Open Source Toolkit for Analysis of Freely-Diffusing Single-Molecule FRET. *PLOS ONE* **11**, e0160716 (2016).
4. Nir, E. *et al.* Shot-Noise Limited Single-Molecule FRET Histograms: Comparison between Theory and Experiments. *J. Phys. Chem. B* **110**, 22103–22124 (2006).
5. Kalinin, S., Valeri, A., Antonik, M., Felekyan, S. & Seidel, C. A. M. Detection of Structural Dynamics by FRET: A Photon Distribution and Fluorescence Lifetime Analysis of Systems with Multiple States. *J. Phys. Chem. B* **114**, 7983–7995 (2010).
6. Antonik, M., Felekyan, S., Gaiduk, A. & Seidel, C. a M. Separating structural heterogeneities from stochastic variations in fluorescence resonance energy transfer distributions via photon distribution analysis. *J. Phys. Chem. B* **110**, 6970–8 (2006).
7. Torella, J. P., Holden, S. J., Santoso, Y., Hohlbein, J. & Kapanidis, A. N. Identifying molecular dynamics in single-molecule FRET experiments with burst variance analysis. *Biophys. J.* **100**, 1568–77 (2011).
8. Tomov, T. E. *et al.* Disentangling Subpopulations in Single-Molecule FRET and ALEX Experiments with Photon Distribution Analysis. *Biophys. J.* **102**, 1163–1173 (2012).
9. Schrimpf, W., Barth, A., Hendrix, J. & Lamb, D. C. PAM: A Framework for Integrated Analysis of Imaging, Single-Molecule, and Ensemble Fluorescence Data. *Biophys. J.* **114**, 1518–1528 (2018).
10. Müller, B. K., Zaychikov, E., Bräuchle, C. & Lamb, D. C. Pulsed Interleaved Excitation. *Biophys. J.* **89**, 3508–3522 (2005).
11. Widengren, J. *et al.* Single-Molecule Detection and Identification of Multiple Species by Multiparameter Fluorescence Detection. *Anal. Chem.* **78**, 2039–2050 (2006).

12. Soranno, A. *et al.* Quantifying internal friction in unfolded and intrinsically disordered proteins with single-molecule spectroscopy. *Proc. Natl. Acad. Sci.* **109**, 17800–17806 (2012).

13. Orte, A., Craggs, T. D., White, S. S., Jackson, S. E. & Klenerman, D. Evidence of an intermediate and parallel pathways in protein unfolding from single-molecule fluorescence. *J. Am. Chem. Soc.* **130**, 7898–7907 (2008).

14. Müller, C. B. *et al.* Precise measurement of diffusion by multi-color dual-focus fluorescence correlation spectroscopy. *EPL Europhys. Lett.* **83**, 46001 (2008).

15. Lapham, J., Rife, J. P., Moore, P. B. & Crothers, D. M. Measurement of diffusion constants for nucleic acids by NMR. *J. Biomol. NMR* **10**, 255–262 (1997).
