# Supplementary material for: The smfBox is an open-source platform for single-molecule FRET
Source: Nat Commun. 2020 Nov 6;11:5641. doi: 10.1038/s41467-020-19468-4 (PMC7648814; doi:10.1038/s41467-020-19468-4)
Supplement: Supplementary file 3 — Description of Additional Supplementary Files [file 41467_2020_19468_MOESM3_ESM.pdf]

Description of Additional Supplementary Files

File Name: Supplementary Data 1
Description: **AutoCAD_Assembled_smfBox.dwg.** An AutoCAD build of the assembled smfBox.

File Name: Supplementary Data 2
Description: **Technical_Drawings_smfBox.dwg.** Technical drawings of all machined parts corresponding to Supplementary Figures 1-14.

File Name: Supplementary Movie 1
Description: **smfBox Animated Build Sequence.** This video provides a fully animated sequence showing all the components of the smfBox coming together in a three-dimensional model.
