# Supplementary material for: The smfBox is an open-source platform for single-molecule FRET
Source: Nat Commun. 2020 Nov 6;11:5641. doi: 10.1038/s41467-020-19468-4 (PMC7648814; doi:10.1038/s41467-020-19468-4)
Supplement: Supplementary file 7 — Reporting Summary [file 41467_2020_19468_MOESM7_ESM.pdf]

nature research

Corresponding author(s): Timothy D. Craggs

Last updated by author(s): 5/10/2020

Reporting Summary

Nature Research wishes to improve the reproducibility of the work that we publish. This form provides structure for consistency and transparency in reporting. For further information on Nature Research policies, see Authors & Referees and the Editorial Policy Checklist.

Statistics

For all statistical analyses, confirm that the following items are present in the figure legend, table legend, main text, or Methods section.

n/a	Confirmed	
☐	☒	The exact sample size (*n*) for each experimental group/condition, given as a discrete number and unit of measurement
☐	☒	A statement on whether measurements were taken from distinct samples or whether the same sample was measured repeatedly
☐	☒	The statistical test(s) used AND whether they are one- or two-sided<br>*Only common tests should be described solely by name; describe more complex techniques in the Methods section.*
☒	☐	A description of all covariates tested
☒	☐	A description of any assumptions or corrections, such as tests of normality and adjustment for multiple comparisons
☐	☒	A full description of the statistical parameters including central tendency (e.g. means) or other basic estimates (e.g. regression coefficient) AND variation (e.g. standard deviation) or associated estimates of uncertainty (e.g. confidence intervals)
☒	☐	For null hypothesis testing, the test statistic (e.g. *F*, *t*, *r*) with confidence intervals, effect sizes, degrees of freedom and *P* value noted<br>*Give P values as exact values whenever suitable.*
☒	☐	For Bayesian analysis, information on the choice of priors and Markov chain Monte Carlo settings
☒	☐	For hierarchical and complex designs, identification of the appropriate level for tests and full reporting of outcomes
☒	☐	Estimates of effect sizes (e.g. Cohen's *d*, Pearson's *r*), indicating how they were calculated

Our web collection on statistics for biologists contains articles on many of the points above.

Software and code

Policy information about availability of computer code

Data collection	Data collected using custom LabVIEW software and C++ software smOTTER available here: https://craggslab.github.io/smfBox/ Thorcam Software version 3.5.1 (64 Bit) was also used in conjunction with the custom LabVIEW code.
Data analysis	Data analysis was performed using custom Jupyter Notebooks running the FRETBursts python module (version 0.7+26.g5de60) (https://fretbursts.readthedocs.io/en/latest/) and with AM (PIE analysis with MATLAB: https://gitlab.com/PAM-PIE/PAM) requiring MATLAB version R2014b (8.4) or newer.

For manuscripts utilizing custom algorithms or software that are central to the research but not yet described in published literature, software must be made available to editors/reviewers. We strongly encourage code deposition in a community repository (e.g. GitHub). See the Nature Research guidelines for submitting code & software for further information.

Data

Policy information about availability of data

All manuscripts must include a data availability statement. This statement should provide the following information, where applicable:

- Accession codes, unique identifiers, or web links for publicly available datasets
- A list of figures that have associated raw data
- A description of any restrictions on data availability

As keen proponents of open science and open data, we have made the photon arrival time data (photon-HDF5 files) which support the findings of this study publicly available from Zenodo, https://zenodo.org/record/3625603#.XxmB3ChKhm9. This includes data pertaining to: Figure 2a – Jnotebooks/definitiveset/1ax.hdf5; Figure 2b/c – Hairpin salt data/1/...; Figure 2d/e – Hairpin hi-mid-low fret data. The individual rate constant determinations for Figure 2c are available in the Source Data file. Full build instructions for the smfBox, including animations, are available through our GitHub https://craggslab.github.io/smfBox/

Field-specific reporting

Please select the one below that is the best fit for your research. If you are not sure, read the appropriate sections before making your selection.

☒ Life sciences ☐ Behavioural & social sciences ☐ Ecological, evolutionary & environmental sciences

For a reference copy of the document with all sections, see nature.com/documents/nr-reporting-summary-flat.pdf

Life sciences study design

All studies must disclose on these points even when the disclosure is negative.

Sample size	Typically, donor and acceptor fluorescent bursts from >1000 molecules were obtained for each smFRET experiment, to ensure sufficient sampling for subsequent analysis. For figure 2e raw data were "cut" into 2000 molecule groups for equal comparison.
Data exclusions	None
Replication	Experiments were generally performed in triplicate, with the exception of the NaCl titration which was performed in duplicate (Fig 2c).
Randomization	Randomization is not applicable to the types of experiments and instrument building described in this manuscript.
Blinding	Blinding was not necessary for the types of experiments ivolved in the biophysical validation of the microscope.

Reporting for specific materials, systems and methods

We require information from authors about some types of materials, experimental systems and methods used in many studies. Here, indicate whether each material, system or method listed is relevant to your study. If you are not sure if a list item applies to your research, read the appropriate section before selecting a response.

Materials & experimental systems

n/a	Involved in the study
☒	☐ Antibodies
☒	☐ Eukaryotic cell lines
☒	☐ Palaeontology
☒	☐ Animals and other organisms
☒	☐ Human research participants
☒	☐ Clinical data

Methods

n/a	Involved in the study
☒	☐ ChIP-seq
☒	☐ Flow cytometry
☒	☐ MRI-based neuroimaging
